# Expression of gynoecium patterning transcription factors in *Aristolochia fimbriata* (Aristolochiaceae) and their contribution to gynostemium development

**DOI:** 10.1186/s13227-020-00149-8

**Published:** 2020-02-17

**Authors:** Pablo Peréz-Mesa, Clara Inés Ortíz-Ramírez, Favio González, Cristina Ferrándiz, Natalia Pabón-Mora

**Affiliations:** 1 Instituto de Biología, Universidad de Antioquia, 050010 Medellín, Colombia; 2 Instituto de Biología Molecular y Celular de Plantas, Consejo Superior de Investigaciones Científicas, Universitat Politècnica de València, 46022 Valencia, Spain; 3 Universidad Nacional de Colombia, Facultad de Ciencias, Instituto de Ciencias Naturales, Sede Bogotá, Colombia

**Keywords:** *CRABS CLAW*, Gynoecium, Gynostemium, *HECATE*, *NGATHA*, *SPATULA*, Stigma, Style

## Abstract

**Background:**

In *Aristolochia* (Aristolochiaceae) flowers, the congenital fusion of the anthers and the commissural, stigmatic lobes forms a gynostemium. Although the molecular bases associated to the apical–basal gynoecium patterning have been described in eudicots, comparative expression studies of the style and stigma regulatory genes have never been performed in early divergent angiosperms possessing a gynostemium.

**Results:**

In this study, we assess the expression of five genes typically involved in gynoecium development in *Aristolochia fimbriata*. We found that all five genes (*AfimCRC*, *AfimSPT*, *AfimNGA*, *AfimHEC1* and *AfimHEC3*) are expressed in the ovary, the placenta, the ovules and the transmitting tract. In addition, only *AfimHEC3*, *AfimNGA* and *AfimSPT* are temporarily expressed during the initiation of the stigma, while none of the genes studied is maintained during the elaboration of the stigmatic surfaces in the gynostemium.

**Conclusions:**

Expression patterns suggest that *CRC*, *HEC*, *NGA* and *SPT* homologs establish ovary and style identity in *Aristolochia fimbriata.* Only *NGA*,*HEC3* and *SPT* genes may play a role in the early differentiation of the stigmatic lobes, but none of the genes studied seems to control late stigma differentiation in the gynostemium. The data gathered so far raises the possibility that such transient expression early on provides sufficient signal for late stigma differentiation or that unidentified late identity genes are controlling stigma development in the gynostemium. Our data does not rule out the possibility that stigmas could correspond to staminal filaments with convergent pollen-receptive surfaces.

## Background

The gynoecium is one of the most complex structures in angiosperms, ensuring proper development, protection and fertilization of the ovules at anthesis and undergoing extreme transformations in the fruit to secure proper seed maturation and dispersal. It is formed by one or more carpels with highly specialized tissues, which represent the fourth and innermost whorl of the flower [1]. The gynoecium is patterned in three major axes: apical–basal, medio-lateral, and adaxial–abaxial, which are determined by specific hormonal and genetic interactions [2]. When two or more carpels are present, they may occur separately (apocarpic) or variously fused together (syncarpic) [1, 3]. Additionally, a typical gynoecium is differentiated from the base to the apex into an ovary usually located at the bottom, carrying the ovules, where fertilization takes place; a style that conducts the male gametophyte(s); and a stigma, which provides a receptive epidermis for landing and germination of the pollen grains [4, 5]. The evolutionary and developmental origins and morphoanatomical innovations shaping the gynoecium are central questions in plant evolutionary biology [6].

The gene regulatory networks involved in gynoecium patterning have been well characterized in *Arabidopsis thaliana* [3, 7–13]. These networks integrate different transcription factor families, hormones, microRNAs, peptides and chromatin-modifying proteins that ultimately define carpel identity and tissue specialization of the gynoecium from fertilization to fruit maturation [6, 14]. Carpel identity is specified by C- and E-function genes, specifically by the tetramer formed between *AGAMOUS* (*AG*) and *SEPALLATA* (*SEP*) MADS-box genes [8, 15–18]. Once carpel identity is acquired, carpel-specific tissues are successively activated (reviewed in [1, 19, 20]). Initially, the *SPATULA* (*SPT*) and *CRABS CLAW* (*CRC*) transcription factors ensure the basal–apical patterning [9]. *CRC* controls carpel growth, apical closure and style development, while *SPT* is required for proper development of the transmitting tissue in the style [9]. Together with *CRC*, the bHLH transcription factors, *SPT* and *HECATE* (*HEC*) are essential for the transmitting tract formation, suppressing the radial growth of the developing gynoecium and promoting its longitudinal growth [11]. This control also reinforces the proximal–distal patterning in syncarpic gynoecia, and the proper differentiation of the style and the stigma by regulating auxin and cytokinin responses [1, 9, 11, 21]. The differentiation of the apical tissues in the gynoecium also requires the expression of *NGATHA* (*NGA*) genes, belonging to the RAV clade of the B3-domain transcription factor family. In *Arabidopsis* the four *NGA* copies act redundantly to direct apical gynoecium development [12, 13]. Similarly to *NGA*, the *SHORT INTERNODES*/*STYLISH* (*SHI*/*STY*) genes, encoding zinc-finger transcription factors, contribute to style and stigma development and proper carpel fusion [10, 22–24]. In *Arabidopsis*, while single mutants in *SHI*/*STY* genes show subtle abnormal formation of the style with no evident fertility loss, the double and multiple mutants show enhanced defects in the style and stigma, similar to those of multiple *NGATHA* mutant combinations. This indicates that these transcription factors work together in a dosage-dependent manner, promoting style and stigma formation during *Arabidopsis* gynoecium development [22, 23].

**Fig. 1:**
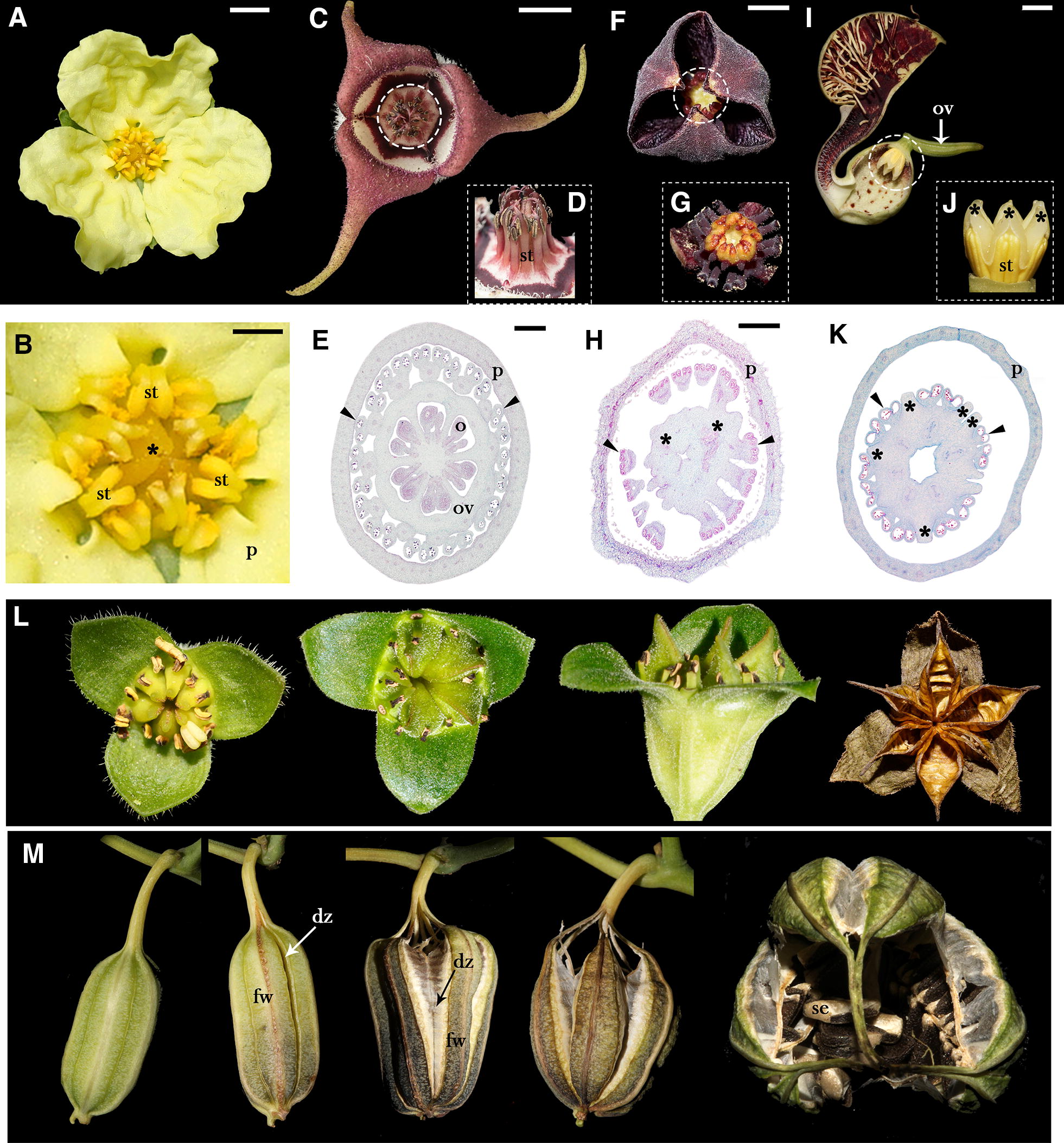
Androecium and gynoecium diversity in Aristolochiaceae. **A**, **B***Saruma henryi,* top view of the flower (**A**) and detail of the 12 stamens in two series free from the six stigmatic lobes (**B**). **C**–**E***Asarum canadense;***C** top view of the flower with 12 stamens in two series free from the six stigmatic lobes. **D** Lateral view of stamens and stigmatic lobes. **E** Transverse section showing the twelve stamens surrounding the 6-carpellate gynoecium. **F**–**H***Thottea siliquosa*; **F** Top view of the Flower. **G** Detail of the 12 stamens surrounding the stigmatic lobes. **H** Transverse section of a preanthetic bud showing the partial fusion between the base of the stamens and stigmas. **I**–**K***Aristolochia fimbriata;***I** young, preanthetic flower. **J** Detail of the gynostemium, lateral view (**J**). **K** Transverse section of the flower showing complete fusion between stamens and stigmatic lobes**. L** Fruit development series in *Saruma henryi.***M** Fruit development series in *Aristolochia fimbriata.* dz, dehiscence zone; fw, fruit wall; o, ovule; ov, ovary; p, perianth; se, seed; st, stamens; asterisks (*) point to stigmas; black arrowheads point to anthers in transverse sections. Scale bars: 1 cm in **A**; 5 mm in **B**–**D**, **F**, **G**, **I**, **J**; 100 μm in **E**, **H** and **K**

Comparative studies suggest that the above-mentioned transcriptional regulators involved in gynoecium patterning have retained similar functions across major flowering plant lineages. Most of the regulatory pathways involved in carpel identity early in the ABCE model (*AG* and *SEP* genes) and later in histogenesis during flower and fruit formation have been maintained over evolutionary time (*CRC*, *NGA, SPT*, or *HEC*) [6, 25–31]. So far the comparative studies of the genetic networks involved in the apical–basal patterning of the carpels have been largely concentrated in the syncarpic gynoecium of *Arabidopsis* and its relatives, but additional data have been obtained from studies in Papaveraceae and grasses, or the single-carpelled gynoecium of legumes [6, 9, 11–13, 29, 32–35]. Here we assess the expression patterns of these transcription factors in *Aristolochia fimbriata*, a species with inferior ovary and an exceptional fusion between stamens and the apices of the carpels forming a gynostemium. The gynostemium is an atypical structure that has evolved independently in a few lineages, including orchids (Orchidaceae: Asparagales), species of *Pauridia* (Hypoxidaceae: Asparagales), *Corsia* (Corsiaceae: Liliales) and in all species of *Aristolochia* (Aristolochiaceae: Piperales) [36, 37]. The gynostemium in *Aristolochia* is formed by the congenital fusion of stamens and stigmas forming a crown-like structure found inside the perianth, above the five or six carpellate, syncarpic, inferior ovary [37]. This feature contrasts with all other closely related perianth-bearing Piperales, as free stamens and stigmas are found in *Asarum* L., *Lactoris* Phil., *Hydnora* Thunb., and *Saruma* Oliver., and an incipient proximal fusion between the stamens and the stigmas is common in *Thottea* Rottb. (Fig. 1) [37–39]. Successful pollen recruitment and fertilization occurs in taxa with free stamens and stigmas, like *Saruma* (Fig. 1L), as well as in those with the gynostemium, like *Aristolochia* (Fig. 1M).

In this work, we characterized the spatio-temporal expression patterns of the *CRC*, *HEC*, *NGA*, and *SPT* homologs in *Aristolochia fimbriata* to understand how these transcription factors are involved in gynostemium development, and how they contribute to the identity of the pollen-receptive gynostemium lobes. We discuss whether shifts from the canonical expression patterns of carpel patterning genes can help to explain the origin and patterning of the gynostemium in this early diverging angiosperm lineage.

## Methods

### Identification of the candidate genes and phylogenetic analyses

The coding sequences of the candidate genes for gynostemium and ovary development were isolated from the *A. fimbriata* transcriptome previously obtained by Pabón-Mora et al. [39], as well as from five newly generated transcriptomes from *Aristolochia arborea*, *A. macrophylla*, *A. manshuriensis*, *Asarum canadense*, *A. europaeum* and *Saruma henryi* (Additional file 1: Table S1). All transcriptomes were generated from mixed leaf, floral and fruit tissues, collected from living collections in the Arnold Arboretum at Harvard University (Additional file 1: Table S1). Tissues were flash frozen in liquid nitrogen. RNA extractions were performed using TRIzol (Invitrogen) following the manufacturers protocol. RNA quality was verified by spectrophotometry in a Nanodrop TM and by electrophoresis in a 1.5% agarose gel. RNA-seq experiments were done by implementing the Truseq mRNA library construction kit (Illumina) and sequenced on a HiSeq2000 instrument reading 100 bases, paired-end reads. Read cleaning was performed with PRINTSEQ-LITE with a quality threshold of Q30 and contig assembly was computed using Trinity package following default settings. The transcriptome assembly was performed for each taxa. Standard metrics for each transcriptome were calculated (Additional file 1: Table S1).

Searches for orthologous genes using the canonical *Arabidopsis* carpel patterning gene sequences and the *Amborella trichopoda* homologs as references were performed using BLASTN [40]. All the orthologous sequences for each gene lineage were compiled using BioEdit (http://www.mbio.ncsu.edu/bioedit/bioedit.html), and manually edited to keep the open reading frame (Additional file 1: Table S2). All nucleotide sequences were then aligned using the online version of MAFFT (http://mafft.cbrc.jp/alignment/server/), with a gap open penalty of 4.0, an offset value of 1,0, and all other default settings. The alignments were then refined manually using BioEdit considering the main domains for each gene lineage. Maximum likelihood (ML) phylogenetic analyses using the full nucleotide coding sequences were performed in RaxML-HPC2 BlackBox [41] on the CIPRES Science Gateway [42]. Bootstrapping was performed according to the default criteria in RAxML, and stopped after 200–600 replicates when the criteria were met. Trees were observed and edited using FigTree v1.4.0 [43]. The new sequences isolated from the transcriptomes of species of *Asarum*, *Aristolochia* and *Saruma* sampled here are available under Genbank numbers: MN709130-MN9154.

### Plant material, RNA isolation and cDNA synthesis

Floral buds at different developmental stages of *Aristolochia fimbriata* were collected from plants cultivated indoors at the Universidad de Antioquia (UdeA). Floral stages 1–10 here used as reference (described in detail by Pabón-Mora et al. [39]) can be summarized as follows: S1 (perianth initiation), S2 (sepal fusion and growth led by the median sepal), S3 (anther primordia and ovary differentiation), S4 (thecae differentiation), S5 (perianth differentiation into utricle, tube and limb and emergence of the stigmatic surfaces adjacent to the anthers), S6 (closure of the limb furrow through interlocking epidermis), S7 (growth of the six stigmatic lobes above the anthers), S8 (resupination of the flower by torsion of the peduncle), S9 (differentiation of the two integuments in the ovules), and S10 (anthesis and expansion of the limb). Total RNA was extracted using TRIzol (Invitrogen, Carlsbad, CA, USA), and treated with DNAseI (Roche, Switzerland) to remove genomic DNA contamination. A total of 3 μg of RNA was used as template for cDNA synthesis with SuperScript III reverse transcriptase (Invitrogen, Carlsbad, CA, USA).

### Reverse transcription PCR (RT-PCR) and quantitative RT-PCR

Apices of flowering shoots and individual floral buds at stages S5, S7 and S9 were used for cDNA synthesis and PCR amplification of *AfimCRC*, *AfimHEC1*, *AfimHEC3*, *AfimNGA*, and *AfimSPT*. PCR assays were done using specific primers (Additional file 1: Table S3), with a thermal cycling regime consisting of one initial step at 94 °C for 10 min, 30 cycles at 94 °C for 40 s, 55 °C for 45 s and 72 °C for 1 min, and a final extension step at 72 °C for 10 min. All reactions were carried out using a MultiGeneTM OptiMax thermocycler (Labnet International, Edison, NJ, USA). The PCR products were run on a 1% agarose gel with 1X TAE, and stained with ethidium bromide, and gels were photographed using a Whatman Biometra^®^ BioDocAnalyzer (Gottingen, Germany).

The quantitative RT-PCR assays were performed from flowers at S5, S7 and S9 using the same protocols for RNA extraction and cDNA synthesis described above. qRT-PCR was done successfully for all genes except the *HEC* homologs, as the similarity between their sequences complicates primer design for short amplicons. The qPCR master mix was prepared using Maxima SYBR Green/ROX qPCR Master Mix K0222 (Waltham, Massachusetts, USA). Two biological replicates and three technical replicates were performed. PCR was done using specific primers (Additional file 1: Table S3), with a thermal cycling regime consisting of one initial step at 95 °C for 3 min, then 40 cycles at 95 °C for 5 s, 54 °C for 5 s, and finally 72 °C for 20 s in a qTOWER^3^ G Real-Time-Thermocycler (Analytik Jena, Jena, Germany). All the target gene expression was analyzed relative to *ACTIN7, ACTIN11,* and *UBIQUITIN* using the 2^−ΔΔC*t*^ method.

### In situ hybridization

In situ hybridizations were performed as described by Ferrándiz et al. [44] with some modifications. *CRC*, *HEC1*, *HEC3, NGA*, and *SPT* DNA templates for RNA antisense and sense probe synthesis were obtained by PCR amplification of 200–400 bp outside of conserved domains defined for each gene lineage (Additional file 1: Table S3). Tissues for hybridization were fixed under vacuum in freshly prepared formaldehyde–acetic acid–ethanol (FAA solution 50% ethanol, 3.7% formaldehyde, and 5% glacial acetic acid) for 2 h. Then, samples were dehydrated in a standard ethanol series, embedded in paraffin and sectioned to 8 μm on a rotary microtome Leica RM2125 RTS. Hybridization was optimized with overnight incubations at 53 °C. Then, sections were washed twice at 53 °C before performing the antibody incubation and the colorimetric reaction. In situ hybridized sections were finally dehydrated and permanently mounted in Permount (Fisher, Waltham, MA, USA). Hybridizations with the sense probes were performed as negative controls. All sections were digitally photographed using the microscopes Nikon Eclipse polarizing e600 equipped with a Leica DM5000 B photographic device.

## Results

### Isolation and expression of carpel identity candidate genes by RT-PCR and qRT-PCR

Homologs of the *CRC*, *SPT*, *NGA*, and *HEC* genes in the studied species of Aristolochiaceae were identified from available mixed floral transcriptomes [39] or newly generated reference transcriptomes described above. Queries used for BLASTN searches included *Arabidopsis* and *Amborella trichopoda* sequences as well as putative homologs from other representative eudicot, monocot and magnoliid species. These searches were first done in the *Aristolochia fimbriata* transcriptome resulting in the identification of one *CRC* gene (named *AfimCRC*), one *SPT* gene (named *AfimSPT* [45]), one *NGA* gene (named *AfimNGA*), and two *HEC* genes (named *AfimHEC1* and *AfimHEC3*). Similar BLAST searches were then performed in the newly generated reference transcriptomes for other species of *Aristolochia*, as well as in *Asarum canadense, A. europaeum* and *Saruma henryi*.

Most genes are found as single copy in all members of the Aristolochiaceae s.l. independently of whether they exhibit free stamens and stigmas or a gynostemium (Additional file 1: Figs. S1–S4). The single copy genes from Aristolochiaceae members predate the duplication events occurring in each gene lineage independently in eudicots and monocots (Additional file 1: Figs. S1–S4). The exception occurs with the *HEC* genes, which have duplicated prior to angiosperm diversification. The two copies found in *A. fimbriata* were named *HEC1* and *HEC3* as phylogenetic analyses assigned them to the *HEC1*/*2* and the *HEC3* clades, respectively [46]. Their homology was assessed in independent phylogenetic analyses for each gene, including sequences representative from each major angiosperm group (Additional file 1: Figs. S1–S4).

**Fig. 2:**
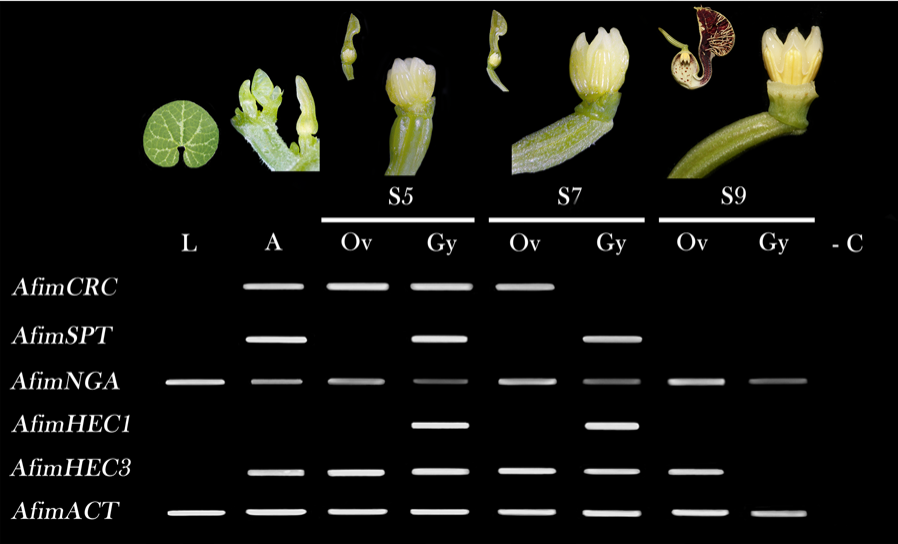
Expression profiles of *AfimCRC*, *AfimSPT*, *AfimNGA*, *AfimHEC1*, and *AfimHEC3* genes at different floral developmental stages, and leaves using standard RT-PCR. Actin (*AfimACT*) was used as a positive control in the RT-PCR. S5, stage 5 of the floral bud, when stigmatic lobes are fully developed. S7, stage 7 of the floral bud, when the stigmatic lobes grow above the anthers and the ovules initiate to develop. S9, stage 9 of the floral bud, when ovules develop the two integuments. L, leaf; A, shoot apex with early floral buds (developmental stages S1–S4); Ov, ovary; Gy, gynostemium; -C indicates the amplification reaction loaded without cDNA

In order to characterize the expression patterns of the five genes putatively involved in the gynostemium and ovary development in *A. fimbriata*, we first used RT-PCR and qRT-PCR (Fig. 2). These results showed that all genes, except *AfimHEC1,* are expressed in the growing flowering shoot (apex), which contains all early flower developmental stages from S1 to S4; Fig. 2). These genes are also found throughout development in the dissected ovary and the gynostemium but only *AfimNGA* is found in leaves (Fig. 2; Additional file 1: Figs. S5). Expression in the dissected ovary and gynostemium across all three floral stages examined (S5, S7 and S9) showed that *AfimCRC* is found in the ovary and in the gynostemium at stage S5, and it is maintained in the ovary at S7, but its expression is no longer detected at stage S9 in the ovary or the gynostemium (Fig. 2; Additional file 1: Fig. S5)*. AfimSPT* is only detected in the gynostemium at stages S5 and S7 (Fig. 2; Additional file 1: Fig. S5). *AfimNGA* is detected at low levels in both ovary and gynostemium at all developmental stages (Fig. 2; Additional file 1: Fig. S5). *AfimHEC1* is detected only in the gynostemium at stages S5 and S7; very low to no expression was detected in the S9 gynostemium or in the ovary at these stages (Fig. 2A). Finally, *AfimHEC3* has a broader expression range compared to its paralog in RT-PCR, with higher expression in both the gynostemium and the ovary at the developmental stages S5 and S7, but it is turned off in the gynostemium at S9 while it is still expressed in the ovary at the same developmental stage (Fig. 2).

### Expression patterns of *AfimCRC* by in situ hybridization

**Fig. 3:**
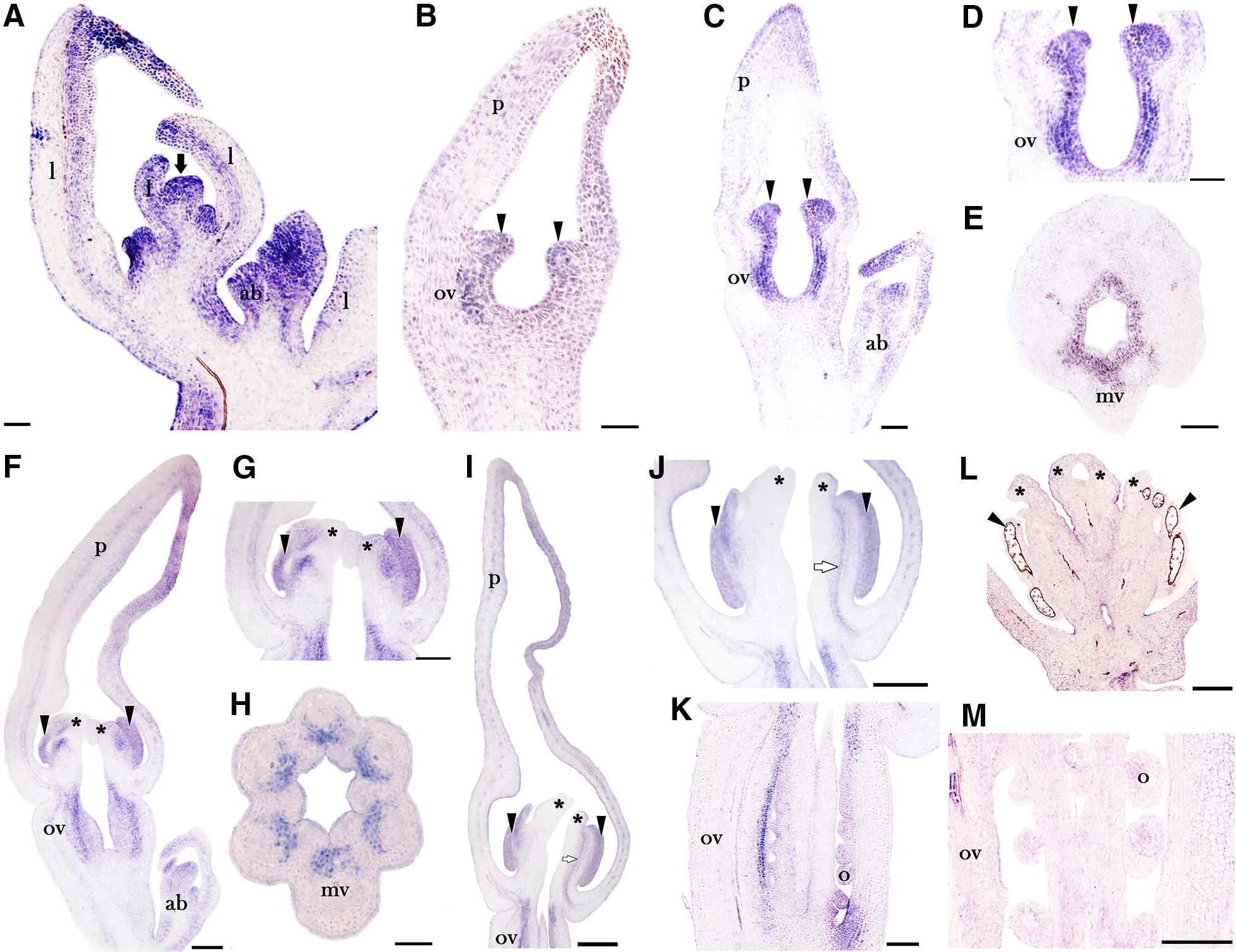
In situ hybridization expression of *AfimCRC*. **A** Longitudinal section of the flowering shoot apex. **B**, **C** Flower bud at stages S3–S4 during stamen formation. **D**, **E** Detailed expression in forming stamens (**D**, longitudinal section) and ovary at S4 (**E**, transverse section)**. F**–**H** Flower at S5 and details of the gynostemium in longitudinal section (**G**), and ovary in transverse section prior to ovule formation (**H**)**. I**–**K** Longitudinal sections of flower at S7 and details of the gynostemium (**J**) and the ovary with the forming ovules in longitudinal section (**K**). **L** Longitudinal section of the gynostemium at S9. **M** Longitudinal section of the ovary with ovules during integument differentiation at S9. ab, accessory bud; l, leaf; mv, midvein of median carpel; o, ovule; ov, ovary; p, perianth. Arrow points to the shoot apical meristem; arrowheads point to stamens or stamen primordia; asterisks (*) point to stigmatic lobes. White arrows (**I**, **J**) show expression of *AfimCRC* in the putative style

All in situ hybridization results are described following the floral developmental stages described by Pabón-Mora et al. [39]. *AfimCRC* is expressed in the shoot apical meristems, the floral primordia (S1–S2), the accessory buds and the adaxial side and distal portion of the young leaves (Fig. 3A). At stages S3 and S4, during the initiation of the anther primordia and the formation of the ovary but prior to stigma initiation, *AfimCRC* is strongly expressed in the stamen primordia and in the ovary (Fig. 3B, C). Expression of *AfimCRC* in the ovary is restricted to the inner epidermis and the 3–5 sub-epidermal cell layers (Fig. 3D, E). At S5, when the stigmatic lobes begin to grow in the adaxial flank of the stamens, *AfimCRC* is detected in the growing stamens, until they reach their boundary with the stigmas, but no expression in the stigmatic lobes of the gynostemium was detected (Fig. 3F, G). The *AfimCRC* expression in the ovary at stage S5 shifts towards the periphery and the mesophyll located to the adaxial side of each vascular bundle (Fig. 3G, H); its expression in the inner epidermal and sub-epidermal layers of the ovary is no longer detected. At stage S7, *AfimCRC* remains expressed in the anthers and in their boundaries with the stigmas during the growth of the stigmatic lobes above the anthers and the ovule initiation (Fig. 3I–K). At this same stage, *AfimCRC* is specifically detected in the transmitting tract, the placenta, the ovary wall and the nucellus of the young ovules (Fig. 3I, K). During S9, *AfimCRC* expression drops dramatically and it is no longer detected in the gynostemium (Fig. 3L). At this stage the ovules develop the two integuments, and *AfimCRC* is only weakly detected in the nucellus (Fig. 3M). Between S1 and S9 *AfimCRC* is also expressed in the distalmost region of the growing perianth (Fig. 3A–C, F–I). Control sense probe for *AfimCRC* resulted in no signal (Additional file 1: Fig. S6).

### Expression patterns of *AfimSPT*

**Fig. 4:**
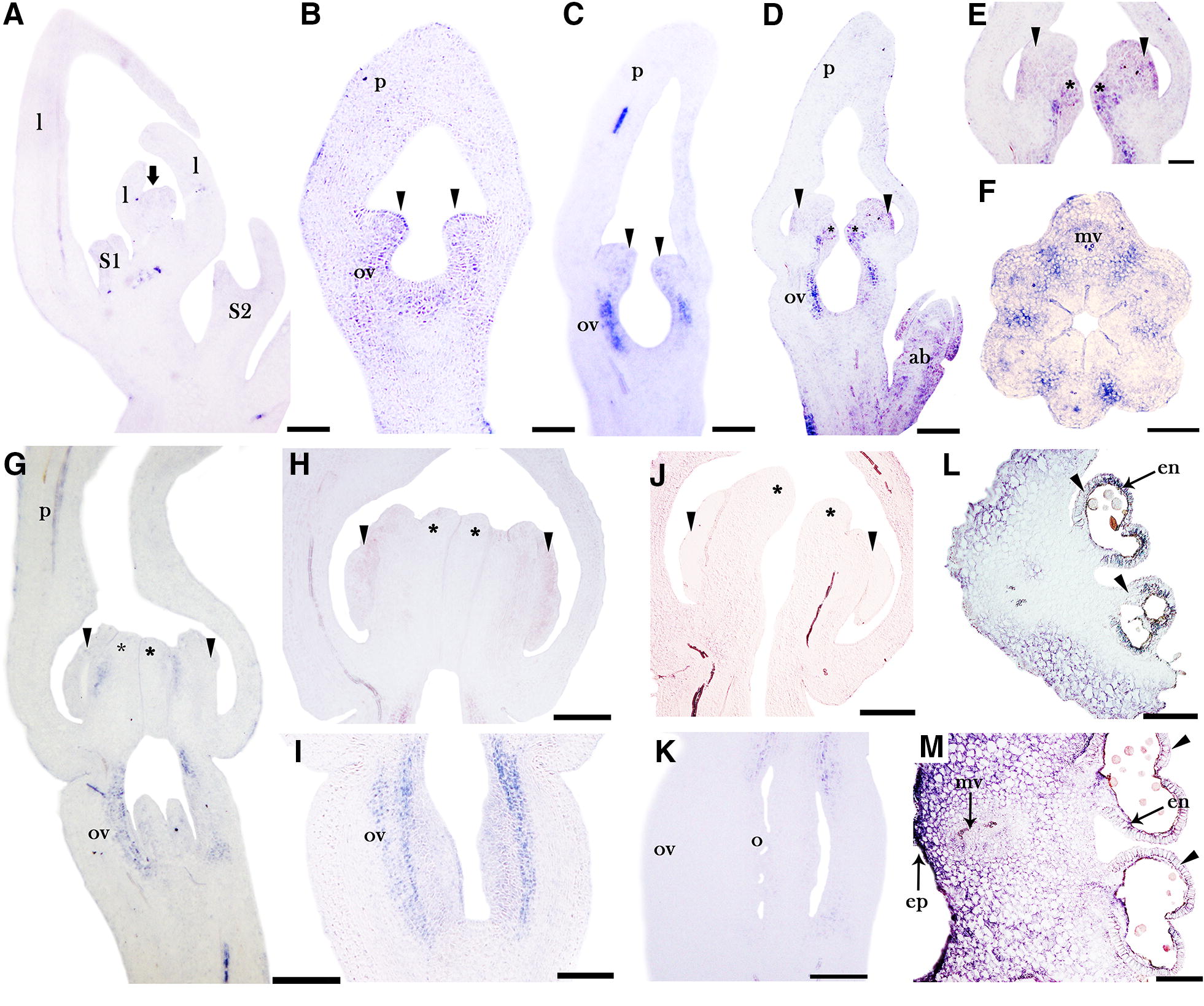
In situ hybridization expression of *AfimSPT*. **A** Flowering shoot apex in longitudinal section. **B**–**D** Successive stages S3–S4 showing initiation of the ovary and stamens. **E**, **F** Longitudinal section of the gynostemium (**E**) and transverse section of the ovary (**F**) at S4. **G**–**I** Flower at S5 with details of the gynostemium with fused stigmatic lobes and anthers (**H**) and ovary in transverse section prior to ovule formation (**I**). **J**, **K** Details of the gynostemium at S7 with the growing stigmas above the anthers (**J**) and ovule differentiation (**K**).** L**, **M** Transverse section of the gynostemium at S9 showing the detail of the fused anthers and stigmas, and the expression at the endothecium, the midvein and the stigmatic epidermis. ab, accessory bud; l, leaf; e, endothecium; ep, stigmatic epidermis; mv, midvein of median carpel; o, ovule; ov, ovary; p, perianth. Arrow points to the shoot apical meristem; arrowheads point to stamens or stamen primordia; asterisks (*) point to stigmatic lobes. Floral stages S1–S9 as in Figs. 2 and 3. Scale bars: 100 μm in **A**, **C**, **F**, **J**–**M**; 50 μm in **B**, **E**; 150 μm in **D**; 200 μm in **G**–**I**)

*AfimSPT* has lower expression levels compared to *AfimCRC*. *AfimSPT* is not detected in the shoot apical meristem, the floral primordia at stages S1 and S2, the accessory buds or the young leaves (Fig. 4A). At stage S3 (stamen initiation and ovary differentiation), *AfimSPT* expression is weakly detected in the stamens and the ovary (Fig. 4B, C). During stage S4, the expression of *AfimSPT* can be detected in the stamens and the ovary, but it is also seen in the forming stigmatic lobes at the adaxial side of the anthers (Fig. 4D, E). Also at S4, *AfimSPT* is detected towards the periphery of the six septal regions of the ovary (Fig. 4F). At stage S5 (when stigmatic tips are fully developed) *AfimSPT* is barely detected in the anthers, and remains only weakly expressed in the ovary, but is no longer expressed in the stigmatic lobes (Fig. 4G–I). During stages S6 and S7, expression is no longer detected in the gynostemium or the ovary (Fig. 4J, K). At S9, the expression of *AfimSPT* is detected in the stamens, specifically in the anther wall (Fig. 4L, M). Unlike *AfimCRC*, *AfimSPT* is not expressed in the perianth during flower development (Fig. 4A–D, G). Control sense probe for *AfimSPT* resulted in no signal (Additional file 1: Fig. S6).

### Expression patterns of *AfimNGA*

**Fig. 5:**
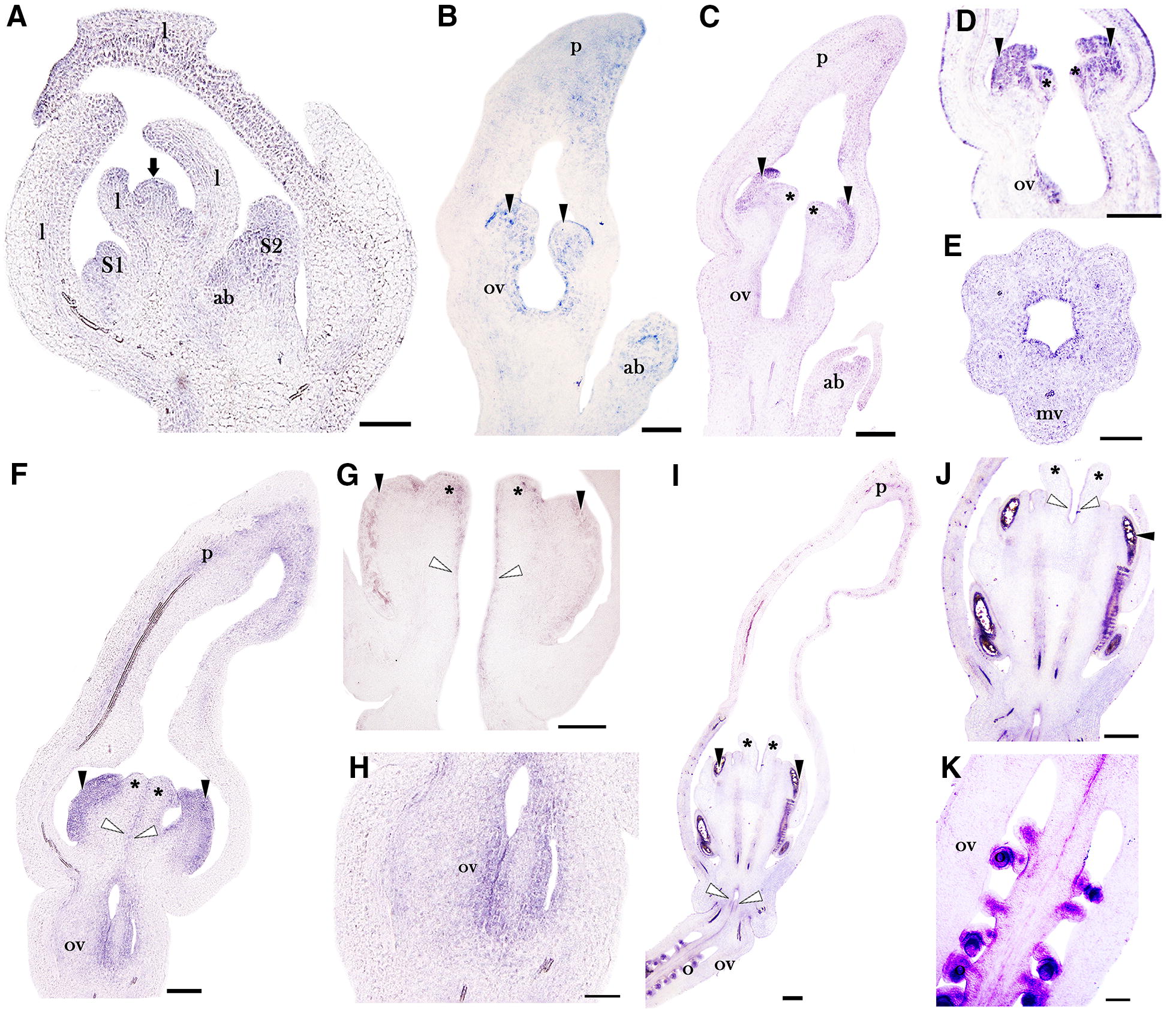
In situ hybridization expression of *AfimNGA*. **A** Flowering shoot apex in longitudinal section. **B** Flower bud at S3 during stamen formation. **C**–**E** Flower at S4 with details of the gynostemium (**D**) and ovary in transverse section (**E**). **F**–**H** Longitudinal sections at S5 with detailed expression in the gynostemium (**G**) and the ovary (**H**). **I**–**K** Longitudinal sections at S7 with detailed expression in the gynostemium (**J**) and the ovary with differentiating ovules (**K**); note in **I** and **J** the anthers overtopped by the stigmas. ab, accessory bud; l, leaf; mv, midvein of median carpel; o, ovule; ov, ovary; p, perianth. Arrow points to the shoot apical meristem; arrowheads point to stamens or stamen primordia; asterisks (*) point to stigmatic lobes. Floral stages S1–S9 as in Figs. 2 and 3. Scale bars: 100 μm (**A**–**I**), 200 μm (**J**), 250 μm (**K**). White arrows (**G**–**J**) show expression of *AfimNGA* in the putative style

The expression of *AfimNGA* is localized in the shoot apical meristem, the S1 and S2 floral primordia, the accessory buds, and the young leaves (Fig. 5A). Its expression at stage S3 is detected in the stamens and the ovary (Fig. 5B), and it is maintained during stages S4 and S5 in the stamens, the developing stigmatic lobes, and the ovary (Fig. 5C–E). In the ovary, expression of *AfimNGA* is restricted to the inner epidermis and the sub-epidermal layers (Fig. 5E). Expression of *AfimNGA* during stage S6 is maintained in the stamens and the ovary while the expression in the stigmas becomes restricted to their elongating tips and the adaxial margin of the stigmatic lobes connecting to the ovary (Fig. 5F–H). At stage S7, when the stigmas grow above the fully differentiated thecae, *AfimNGA* is expressed in the developing ovules, the septal regions of the ovary, the adaxial margins of the stigmatic lobes, the sporogenous tissue in the anthers, and the pollen grains (Fig. 5I–K). Later, stages S8 and S9, when ovules develop the two integuments, the signal of *AfimNGA* is found in the nucellus, the integuments, and the epidermal cells in the transmitting tract (Fig. 5J–K). *AfimNGA* is also expressed in the growing perianth during flower development (Fig. 5A–C, F, I). Control sense probe for *AfimNGA* resulted in no signal (Additional file 1: Fig. S6).

### Expression patterns of *AfimHEC1* and *AfimHEC3*

**Fig. 6:**
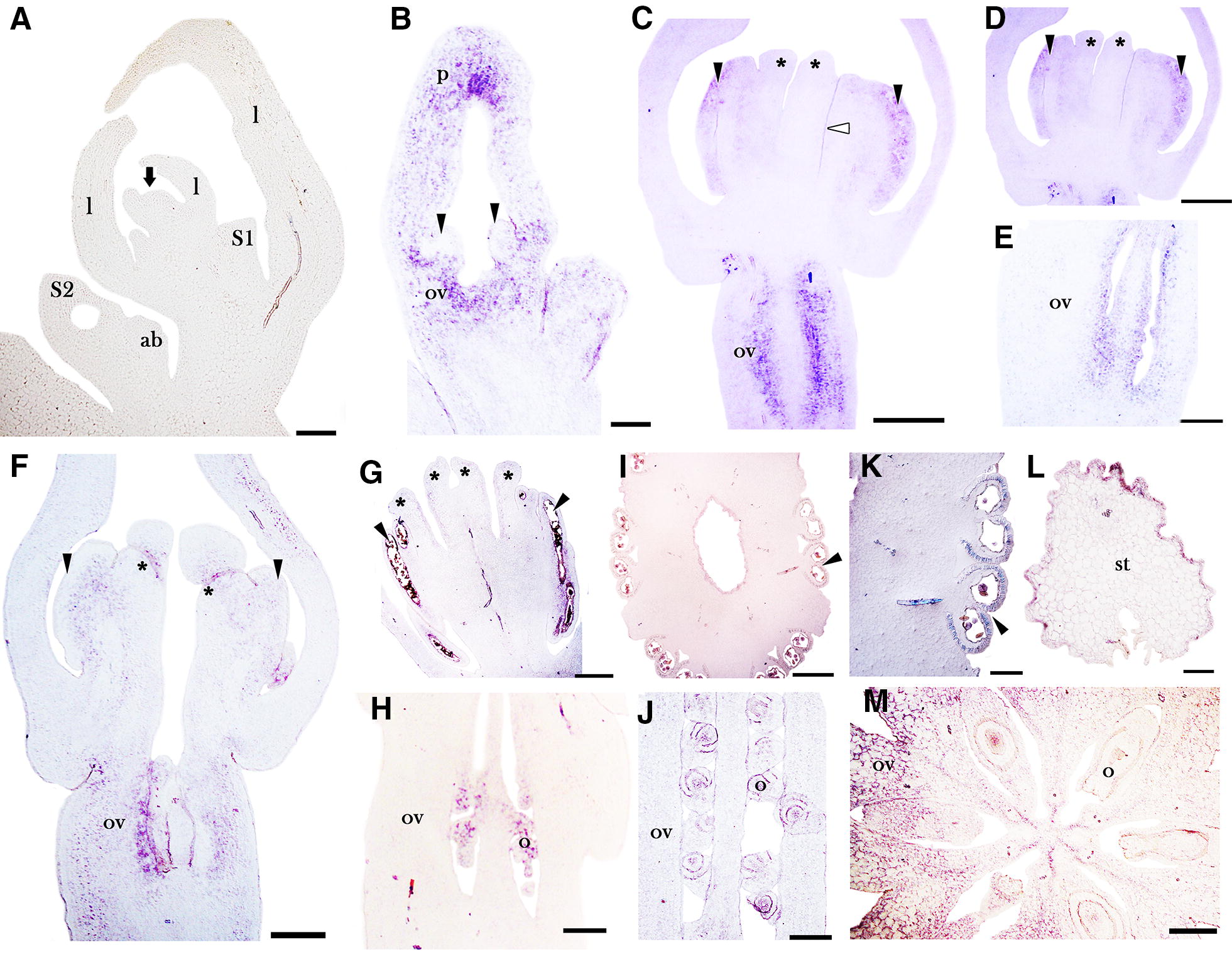
In situ hybridization expression of *AfimHEC1*. **A** Longitudinal section of the flowering shoot apex. **B** Longitudinal section of the floral bud at S3. **C-E** Longitudinal sections at S5 showing expression in stamens (**C**, **D**) and ovary (**E**). **F**–**H** Longitudinal section of the floral bud at S7, with expression in the gynostemium (**F**, **G**) and ovary during ovule initiation (**H**). **I** Transverse section of the gynostemium at S9. **J** Longitudinal section of the ovary showing the developing ovules at S9. **K** Detail of a tetrasporangiate anther fused to a stigmatic lobe at S9. **L** Expression at S9 in preanthesis of the stigmatic epidermis. **M** Transverse section of the ovary during ovule-to-seed transition. ab, accessory bud; l, leaf; mv, midvein of median carpel; o, ovule; ov, ovary; p, perianth. Arrow points to the shoot apical meristem; arrowheads point to stamens or stamen primordia; asterisks (*) point to stigmatic lobes. Floral stages S1–S9 as in Figs. 2 and 3. Scale bars: 100 μm in **A**, **C**–**E**, **I**; 50 μm in **B**, **K**, **L**; 200 μm in **F**–**H**; 150 μm in **J**, **M**. White arrowheads (**C**) indicate expression in the junction between stamens and stigmatic lobes

*AfimHEC1* is not detected at the shoot apical meristem, the floral primordia at S1 and S2, the accessory buds or the young leaves (Fig. 6A). During S3, *AfimHEC1* is first detected in the ovary and the distal portions of the perianth and it is not detected in the incipient anther primordia (Fig. 6B). During S5, when stigmatic lobes are differentiated, the expression of *AfimHEC1* is maintained in the ovary and in the stamens but not in the stigmatic lobes (Fig. 6C–E). During S6 and S7, *AfimHEC1* expression is maintained in the stamens, the ovary, and the young ovules (Fig. 6F–H). At stages S7 and S9, the expression of *AfimHEC1* is mostly restricted to the nucellus and the integuments of the developing ovules as well as in the pollen grains (Fig. 6I–K). Expression at S9 in preanthesis, can be seen only in the epidermis of the stigmatic lobes (Fig. 6L). During the ovule-to-seed transition *AfimHEC1* expression is weakly detected in the nucellus remnants, but lacking in the integuments or the seed coat (Fig. 6M).

**Fig. 7:**
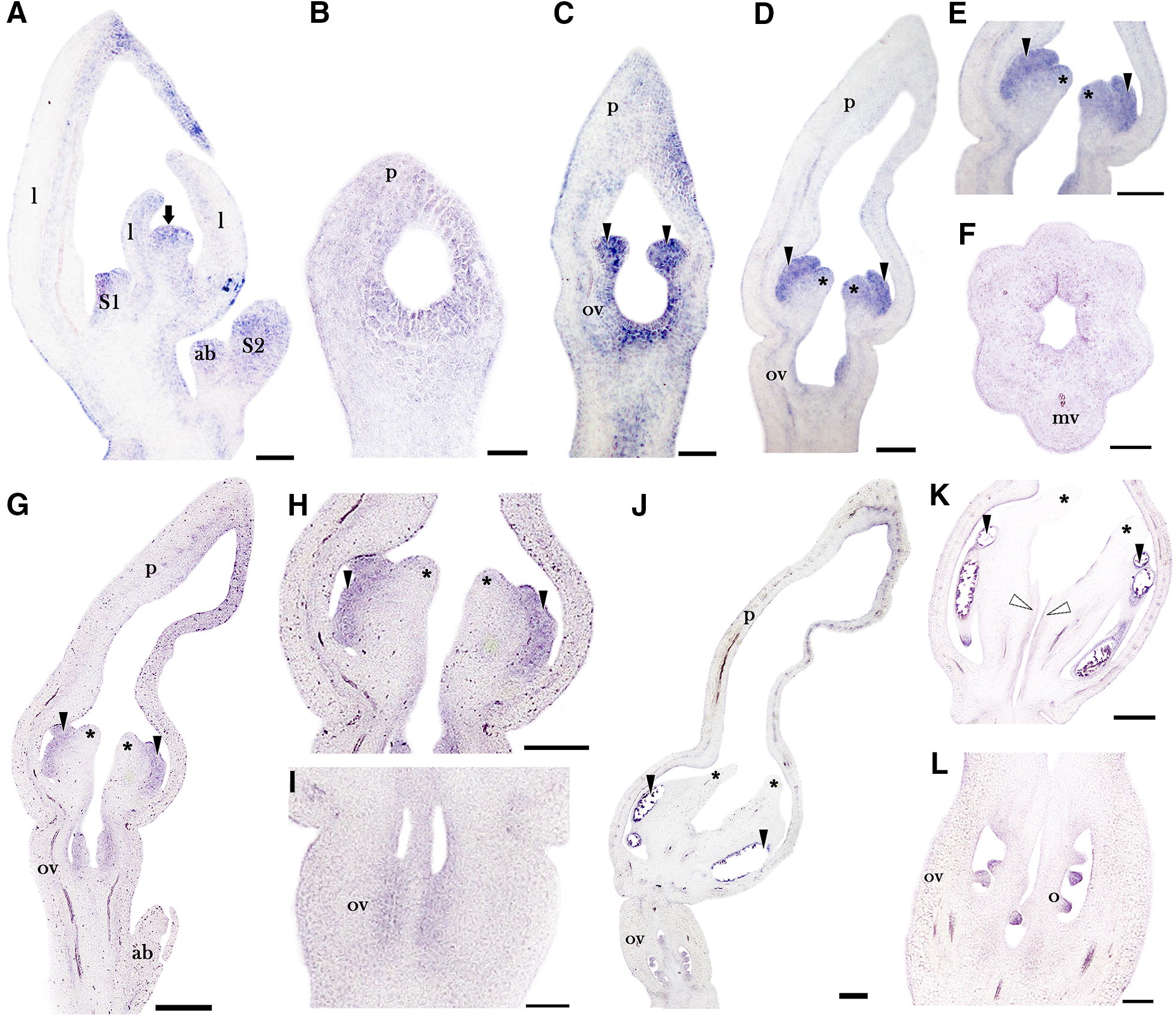
In situ hybridization expression of *AfimHEC3*. **A** Longitudinal section of the flowering shoot apex. **B** Flower bud at S2, longitudinal section. **C** Floral bud at S3 during stamen initiation, longitudinal section. **D**–**F** Flower at S4 with details of the expression in stamens and stigmas (**D**, **E**) in longitudinal section, and ovary in transverse section (**F**). **G**–**I** Longitudinal section of a S5 floral bud with detailed gynostemium (**H**) and ovary (**I**). **J**–**L** Longitudinal sections of a S7 floral bud with detailed gynostemium (**K**) and ovary during ovule initiation (**L**). ab, accessory bud; l, leaf; mv, midvein of median carpel; o, ovule; ov, ovary; p, perianth. Arrow points to the shoot apical meristem; arrowheads point to stamens or stamen primordia; asterisks (*) point to stigmatic lobes. Floral stages S1–S9 as in Figs. 2 and 3. Scale bars: 100 μm in **A**, **C**–**F**, **J**; 50 μm in **B**; 250 μm in **G**; 150 μm in **H**, **I**; 200 μm in **K**, **L**. White arrowheads (**K**) indicate expression in the putative style

In general, the expression patterns of *AfimHEC3* are stronger and broader than that of *AfimHEC1* in all the stages analyzed (Figs. 6 and 7). Expression of *AfimHEC3* is first detected in the shoot apical meristem, the young leaves, the young S1 and S2 flowers, and the accessory buds (Fig. 7A, B). At stage S3, *AfimHEC3* is strongly expressed in the developing stamens and the ovary (Fig. 7C). At stage S4 (stigma initiation), the expression is detected in the stamens, the developing stigmas, and the ovary (Fig. 7D–F). Expression in the ovary is mostly detected in the inner epidermis (Fig. 7D, F). At stage S5, when stigmas are fully differentiated and reach the same size as the stamens, *AfimHEC3* is still detected in the stamens and the ovary, but it is no longer evident in the stigmatic lobes (Fig. 7G–I). At stage S7, when the stigmatic lobes overtop the stamens, expression of *AfimHEC3* is retained only at the sporogenous anther tissue, the ovules and the transmitting tract (Fig. 7J–L). Control sense probes for *AfimHEC* paralogs resulted in no signal (Additional file 1: Fig. S6).

## Discussion

The gynoecium often differentiates into the proximal ovary, and the distal style and stigma. Distal portions are highly specialized being primarily responsible for pre-zygotic selection of pollen grains [1, 47]. *Aristolochia* flowers provide an ideal system to study whether the genetic networks shaping the carpel patterning are retained in highly modified gynoecia, with stigmas fused to the sessile anthers forming a gynostemium. Although the gynostemium lobes are functionally stigmatic, their odd position opposite to the stamens and alternate to the carpels (as commissural structures) has led to an alternative interpretation as massive filaments rather than as true gynoecium-derived tissue, or a combination of both, with stamen identity retained externally and stigma identity retained internally (see review in [37]). Despite the occurrence of congenital fusion between stigmas and stamens [37], the flower exhibits an intricate cross-pollination system where proterogyny avoids selfing. Stigmas mature first and become wet, expanded and receptive to pollen grains, which need to germinate and pass through the stigmatic and stylar regions before the perianth and the gynostemium detaches from the inferior ovary [39, 48].

Having as a reference point previously identified key transcription factors in *Arabidopsis* involved in shaping and patterning of the gynoecium, we have studied here the expression patterns of *CRC*, *SPT*, *NGA*, and *HEC* homologs in *Aristolochia fimbriata.* We aim to understand what genes are at the core of gynoecium patterning despite such extreme modifications in a phylogenetically distant early diverging angiosperm. In addition, we also look to identify whether the expression of these genes can be linked to the identity and elaboration of the stigmatic lobes in the highly elaborated gynostemium. All the predicted roles based on expression patterns will have to be confirmed in the future with functional analyses when available for this non-model species.

### Expression of *AfimCRC* is likely correlated with the receptacular inferior ovary in *Aristolochia fimbriata*

The *CRC* gene belongs to the YABBY family of transcription factors present in the last common ancestor of all angiosperms [49, 50]. Expression patterns and functional analyses in most species studied suggest conserved roles of *CRC* homologs in the differentiation of the stigma and the style, the fusion of the carpel margins, proper gynoecium medial–lateral patterning, and only in some cases, the development of nectaries and the termination of the floral meristem [33–35, 49, 51–56]. In eudicots like *Arabidopsis*, *Petunia hybrida*, *Nicotiana benthamiana*, and *Eschscholzia californica*, *CRC* homologs are mostly expressed in the carpel primordia, the abaxial region of the gynoecium, and the developing nectaries [9, 51, 53, 55, 56]. However, *CRC* homologs in grasses and in pea lack a clear abaxial expression domain and appear rather homogenously expressed in the carpels [33, 35]. The *crc* mutants in core eudicots show abnormally wider gynoecia with unfused carpels, and loss of nectaries [5, 9, 51, 53, 55, 56]. In species of the monocot families Asparagaceae and Poaceae, as well as in *Pisum sativum* (Leguminosae) and *Eschscholzia californica* (Papaveraceae) *CRC*-like genes control carpel morphology and vasculature [33–35, 55, 57]. *CRC* genes are also responsible for floral meristem termination in eudicots and monocots [33, 55, 56]. In the case of *Arabidopsis*, the role of *CRC* in meristem determinacy is masked by redundant pathways with *AGAMOUS* and only *crc*-*1 ag*-*1*/+ and *crc*-*1 ap2*-*2 pi*-*1 ag*-*1* mutants show abnormal carpel proliferation in the center of the flower [5, 9, 14, 33, 55, 56].

Less is known about the role of *CRC*-like genes in early divergent angiosperms. However, expression studies of *CRC* homologs in *Amborella trichopoda* (Amborellaceae) and *Cabomba caroliniana* (Cabombaceae) show conserved expression patterns in abaxial tissues of the developing carpels [49, 52]. However, in these species, *CRC* homologs are also found in the floral apex, the perianth, and the stamens, indicating broader ancestral roles for *CRC* homologs that could have been lost before the divergence of monocots and eudicots [49]. Our results support this idea, as expression patterns of *AfimCRC* in *Aristolochia* resemble those documented in *A. trichopoda* and *C. caroliniana*, in the developing flowering shoot apex, the perianth at different developmental stages, the anthers, the ovary and its vascular tissue, the ovules, and the young leaves (Fig. 3). However, a noticeable difference when compared to the expression recorded for *CRC* homologs in all other angiosperms is the apparent shift of *AfimCRC* expression to the adaxial surfaces of the ovary in *A. fimbriata*. Unlike all other angiosperms with recorded *CRC* expression, *A. fimbriata* is the only one with inferior ovary, which is congenitally surrounded by the floral receptacle, resulting in a composite axial–carpellary tissue. Thus, our results point to *AfimCRC* expression as a marker for carpel-derived tissue on the inside of this complex structure. However, these hypotheses need to be corroborated with studies in other species with inferior ovary. Our findings in *A*. *fimbriata,* together with previous reports, suggest that the plesiomorphic role of *CRC* homologs include vascular differentiation in the carpels, a function maintained in other eudicots and monocots [33, 35, 55]. Also, *AfimCRC* expression in the inside of the ovary suggests an ancestral role in the specialization of the inner layers for placenta development and ovule initiation, shared, at least, with basal eudicots [55]. Finally, it is also possible that *AfimCRC* can contribute to the boundary establishment and maintenance between stamens and stigmatic lobes and the development of the transmitting tissue in *A. fimbriata*.

Conversely, the results presented for *AfimCRC* do not support its putative contribution for stigma and style development, suggesting two possible scenarios: (1) there are other transcriptional regulators controlling the identity of the apical carpel tissues, or (2) the expression detected in the ovary and the stamens could be associated with a putative non-cell-autonomous activity of *AfimCRC*¸ regulating in this case the activation of additional transcription factors that promote stigma identity, as it has been proposed in other eudicot species [35, 55].

### Reduced levels of *AfimSPT* in the stigmas are only found transiently early during the gynostemium development of *Aristolochia fimbriata*

The *SPT* gene encodes a bHLH transcription factor present in both gymnosperms and angiosperms [45, 58, 59]. *SPT* is one of the two copies that, along with its paralog *ALCATRAZ* (*ALC*), resulted from a core eudicot duplication event. Both genes are involved in gynoecium and fruit patterning in angiosperms. In *Arabidopsis thaliana, SPT* promotes the development of specialized tissues of the carpel margins, and regulates the differentiation of the style, the stigma, and the transmitting tissue [5, 9, 60]. Additionally, *SPT* specifies the differentiation of the valve margins and the dehiscence zone during carpel development and, together with *INDEHISCENT* (*IND*), it also regulates the auxin biosynthesis and distribution in the medial tissues during development [14, 61, 62]. Expression of *SPT* homologs in species of Solanaceae is broad in sepals, petals, stamens and carpels [63]. Functional analyses suggest that, together with *ALC*, *SPT* genes promote cell division and organ size as well as fruit maturation and ripening, likely by repressing lignification [63]. Similar broad expression patterns in the perianth, the ovary and the lignified layer of the fruits (endocarp) has been found for *SPT/ALC* homologs in peach (*Prunus persica,* Rosaceae), and it has been suggested that they function in endocarp differentiation [64]. Expression studies of the paleo *SPT*/*ALC* in basal eudicots such as *Bocconia frutescens* (Papaveraceae) have shown that *BofrSPT* homologs have broad expression patterns in sepals, stamens, as well as in the medial fusion zone of the carpels, the growing ovules, and the dehiscence zone during fruit development [45]. Our results in *A*. *fimbriata* are different compared with those described above in eudicot species. *AfimSPT* expression is lacking at style and stigma initiation stages, and its expression is mostly restricted to the ovary at specific developmental stages (Fig. 4C, D, F, G). *AfimSPT* is only weakly detected in the stigma primordia in early developmental stages, so it cannot be disregarded as an initial signal for other regulators to control the differentiation of the apical specialized tissues (Fig. 4D–E). However, *AfimSPT* expression is not maintained during style and stigmatic lobe differentiation. Thus, considering that *SPT* homologs promote fusion between stigmatic lobes, the low levels of *AfimSPT* can be associated with the separation of the stigmatic tips in the gynostemium. In addition, the expression reported here in the ovary wall of *Aristolochia* could be associated with the longitudinal growth of the gynoecium during flower development and the formation of the dehiscence zone later on during fruit maturation (Fig. 1M), as it has been reported in other eudicot species like *A*. *thaliana* and *B*. *frutescens* [5, 9, 45]. Overall, these findings suggest that the expression patterns of *AfimSPT* differ from that previously reported for eudicot species, except for the expression found in the ovary walls, indicating only conserved roles for the putative dehiscence zone formation during fruit development.

### The expression pattern of *AfimNGA* is linked to style/stigma development and differentiation in *Aristolochia fimbriata*

The *NGA* genes are members of the B3 transcription factor family in angiosperms; the four *Arabidopsis* paralogs result from Brassicaceae-specific duplication events [12, 13]. Functional data from *Arabidopsis* indicate that all four copies act redundantly to control style and stigma fusion and development [12, 13, 63, 65]. The same functions have been identified for the *NGA* homologs in *Eschscholzia californica* and *Nicotiana benthamiana*. Down-regulation of *EcNGA* and *NbNGA* genes results in severe defects including opened styles and reduced stigmatic tissues [6]. Based on the available data, it has been proposed that *NGA* genes have conserved roles in style and stigma development in eudicots [6]. *NGA* genes have not been evaluated in monocots, early diverging angiosperms or gymnosperms, thus our expression studies are the first to assess the putative contribution of *NGA* genes to gynoecium development outside eudicots. Our data show that *AfimNGA* is expressed in the stamen primordia, the growing stigmatic lobes at early developmental stages, the inner epidermis of the ovary, the medial zone of the short style, and the ovules at late developmental stages (Fig. 5). These patterns suggest that *AfimNGA* functions in the early stigma identity, similar to what has been reported in eudicot *NGA* homologs, but later on, it becomes restricted to the lining of the stigma and the putative style in the modified gynostemium of *Aristolochia*. It is possible that a limited expression of *AfimNGA* in concert with a low expression of *AfimSPT* result in the separation of the stigmatic lobes during gynostemium development. Importantly, *AfimNGA* is present in the short style, in the ovary, and in the ovules, suggesting that its role in the specification of the transmitting tract tissue remains intact even in the absence of a fully fused stigma, possibly allowing the growth of the pollen tubes along the style and into the ovary. Overall, these results suggest that *AfimNGA* is likely to be one of the essential regulators of the style and stigma development in *Aristolochia*, a function shared by early divergent angiosperms and eudicots studied so far. It remains to be tested whether *AfimNGA* is contributing in the auxin signaling in the gynostemium by the interaction with additional regulatory factors, as it occurs in *Arabidopsis* [10, 13, 23, 66–68].

### *AfimHEC3* regulates the identity of the style and the stigma while *AfimHEC1* contributes to ovary development in *Aristolochia fimbriata*

The bHLH *HEC1*-*3* transcription factors control multiple developmental processes like shoot meristem activity and auxin signaling in *Arabidopsis* [11, 21, 69]. Also, *HEC* genes appear to coordinate in a partially redundant manner the transmitting tract development [11]. The *hec1*,*2*,*3* triple mutants in *Arabidopsis* show severe defects in stigma and transmitting tissues, and milder defects in style and septum fusion resulting in complete fertility loss [11]. In other eudicots like in Solanaceae species, the expression data available suggest that only *HEC3* genes are involved in gynoecium patterning, while *HEC1/2* transcription factors are more likely associated with fruit maturation [59]. In our study, *AfimHEC1* is detected in the ovary and in the stamens (Fig. 6) while *AfimHEC3* is specifically detected in the stigmas, the stamens, the style, the mature ovules, and weakly detected in the ovary (Fig. 7). These differential expression patterns detected for the two *AfimHEC* genes in *Aristolochia fimbriata* differ from the redundant expression patterns found in the *HEC* homologs of *Arabidopsis* in the septum, the transmitting tract, and the stigma [11]. *AfimHEC3* is detected in the stigmatic tips and the medial zone of the style (Fig. 7K), suggesting that together with *AfimNGA,* can control the differentiation of the apical specialized tissues of the carpels, and the formation of a transmitting tract for pollen tube growth to the ovules during the fertilization. Moreover, *AfimHEC1* expression suggests functions associated with ovary and fruit development, more similar to what has been described for species of Solanaceae, indicating alternative roles of the *AfimHEC* genes during flower and fruit development.

### Putative genetic mechanisms involved in the gynostemium development of *Aristolochia fimbriata*

Gynoecium development requires multiple biological regulators that control ovule formation and protection as well as the transformation of specialized tissues especially in response to fertilization and during fruit patterning. Multiple regulatory networks have been proposed to describe the different developmental processes that occur from floral meristem initiation until fruit maturation and seed release (reviewed in Zuñiga-Mayo et al. [14]). However, little information on the function of these genes is available in early diverging flowering plants and especially in those with unconventional stamen–stigma patterning forming a gynostemium. The gynostemium, as we previously described, is rare in flowering plants, and the fusion between the stigmas and stamens presents an excellent opportunity to study signaling pathways that control congenital organ fusion. In this work, the expression patterns of the candidate transcription factors evaluated for gynostemium development suggest a direct contribution to stamen, ovary and ovule differentiation for all *AfimCRC*, *AfimSPT, AfimNGA* and *AfimHEC* genes. *AfimCRC* also specifies carpel domains in the inferior ovary surrounded by receptacular tissue. However, only *AfimSPT, AfimNGA* and *AfimHEC3* genes are actively participating in early stigma identity early on, although rather transiently, and in style differentiation later in development, especially in those domains where transmitting tissue will develop. In addition, and based on their overlapping expression, it is possible to speculate that these same three genes, together with *AfimCRC,* act maintaining the gynoecia boundaries inside the gynostemium throughout development, given that they are retained in the abaxial flanks of the stigmas even though their expression is not maintained in the stigmatic tips. Finally, the promotion of stigmatic features like the development of papillae during late stages of development and the secretion and proliferation of exudates during the female phase of the flower does not appear to depend on the canonical stigmatic genes described so far, and other genes with late activation may control such features. It is also possible that stigmatic tips in the gynostemium are in fact the result of convergent features derived from the stamens. However, the B and C class MADS-box genes that confer stamen identity have not been found to be expressed in the stigmatic tips either [39, 70], suggesting that also in this scenario, late unidentified genes may be major players in stigmatic differentiation.

## Conclusion

From our study we can conclude that the gynostemium lobes in *Aristolochia* flowers are functionally stigmatic, however, their odd position opposite to the stamens and alternate to the carpels (as commissural structures), as well as the lack of continuous expression of canonical style–stigma genes supports two alternative scenarios: one, where the stigmatic tips are still gynoecium-derived but unidentified genes with late expression control stigma differentiation and elaboration; the other, where the gynostemium as formed by the proliferation of massive filaments externally and true gynoecium identity restricted to the transmitting tissue tract. The three genes that are most likely to turn on style–stigma identity early on, inside of the *Aristolochia* gynostemium are *AfimSPT, AfimNGA* and *AfimHEC3*; however, their expression is transient in early stages and may not control late identity of pollen-receptive surfaces.

## Supplementary information

**Additional file 1: Table** **1.** Plant collections, locations, and statistics from the TRINITY assembly of mixed transcriptomes obtained for each of the members of the perianth-bearing Piperales. **Table S2.** Accession numbers of *CRABS CLAW, SPATULA, NGATHA, and HECATE* homolog sequences used in this study. **Table S3.** Primers used in this study. Gene specific primers used in this study to amplify *AfimCRC, AfimSPT, AfimNGA*, and *AfimHEC1,* and *AfimHEC3* orthologs in *Aristolochia fimbriata*, using reverse transcription polymerase chain reaction (RT-PCR), quantitative reverse transcription (qRT-PCR), and in situ hybridization (ISH) experiments. **Figure S1.** Maximum likelihood (ML) phylogenetic analysis of the *CRC* gene lineage in flowering plants. The outgroup used was the *Amborella trichopoda AmtrCRC.* Bootstrap (BS) supports shown above the nodes. Colors follow conventions on the figure. **Figure S2.** Maximum likelihood (ML) phylogenetic analysis of the *SPT* gene lineage in flowering plants. Outgroup used was *Cycas micholitzii SPT.* Star points to a duplication event. BS supports shown in the nodes. Colors follow conventions on the figure. **Figure S3.** Maximum likelihood (ML) phylogenetic analysis of the *NGA* gene lineage in flowering plants. The outgroup used was *Arabidopsis thaliana RAV.* Star points to a duplication event. BS supports shown in the nodes. Colors follow conventions on the figure. **Figure S4.** Maximum likelihood (ML) phylogenetic analysis of the *HEC* gene lineage in flowering plants. Outgroup used was *Arabidopsis thaliana bHLH87.* Star points to a duplication event. BS supports shown in the nodes. Colors follow conventions on the figure. **Figure S5.** Relative expression profiles of *AfimCRC*, *AfimSPT* and *AfimNGA* at different floral developmental stages, and leaves using standard qRT-PCR. Ubiquitin (*AfimUBC*) was used as a positive control. **Figure S6.** In situ hybridization with sense probes for all tested genes in *Aristolochia fimbriata*.

## Data Availability

All supporting data are available in the Additional files.

## Abbreviations

AG: AGAMOUS
ALC: ALCATRAZ
bHLH: Basic/helix–loop–helix
CRC: CRABS CLAW
HEC: HECATE
IND: INDEHISCENT
ML: Maximum likelihood
NGA: NGATHA
qRT-PCR: Quantitative polymerase chain reaction
RT-PCR: Reverse transcription polymerase chain reaction
SEP: SEPALLATA
SPT: SPATULA
STY: STYLISH
