# Supplementary material for: Expression of gynoecium patterning transcription factors in Aristolochia fimbriata (Aristolochiaceae) and their contribution to gynostemium development
Source: EvoDevo. 2020 Feb 17;11:4. doi: 10.1186/s13227-020-00149-8 (PMC7027301; doi:10.1186/s13227-020-00149-8)

Additional File 1

Table S1. Plant collections, locations, and statistics from the TRINITY assembly of mixed transcriptomes obtained for each of the members of the perianth-bearing Piperales.

Species	Collection location	Trinity statistics
Asarum canadense	Living collections at the Arnold Arboretum at Harvard University (Roslindale, MA, USA). Plant ID: 204-2006*MASS-A	Total length of sequence: 106980895 bp<br>Total number of sequences: 103891<br>Average contig length is: 1029 bp<br>Largest contig: 16629 bp<br>Shortest contig: 201 bp<br>N25 stats: 25% of total sequence length is contained in the 7278 sequences >= 2696 bp<br>N50 stats: 50% of total sequence length is contained in the 19765 sequences >= 1738 bp<br>N75 stats: 75% of total sequence length is contained in the 40296 sequences >= 934 bp<br>Total GC count: 46257633 bp<br>GC %: 43.24 %
Asarum europaeum	Living collections at the Arnold Arboretum at Harvard University (Roslindale, MA, USA). Plant ID: 202-2006*MASS	Total length of sequence: 89804434 bp<br>Total number of sequences: 81419<br>Average contig length is: 1102 bp<br>Largest contig: 16268 bp<br>Shortest contig: 201 bp<br>N25 stats: 25% of total sequence length is contained in the 5951 sequences >= 2747 bp<br>N50 stats: 50% of total sequence length is contained in the 16149 sequences >= 1811 bp<br>N75 stats: 75% of total sequence length is contained in the 32230 sequences >= 1040 bp<br>Total GC count: 38999723 bp<br>GC %: 43.43 %
Aristolochia arborea	Living collections at the New York Botanical Garden. Enid A Haupt Conservatory (Bronx, NY, USA).	
Aristolochia macrophylla	Living collections at the Arnold Arboretum at Harvard University (Roslindale, MA, USA). Plant ID: 1112-88*A	Total length of sequence: 164714213 bp<br>Total number of sequences: 126783<br>Average contig length is: 1299 bp<br>Largest contig: 15711 bp<br>Shortest contig: 201 bp<br>N25 stats: 25% of total sequence length is contained in the 9747 sequences >= 3158 bp

		N50 stats: 50% of total sequence length is contained in the 25812 sequences >= 2127 bp<br>N75 stats: 75% of total sequence length is contained in the 50215 sequences >= 1295 bp<br>Total GC count: 71235570 bp<br>GC %: 43.25 %
Aristolochia manshuriensis	Living collections at the Arnold Arboretum at Harvard University (Roslindale, MA, USA). Plant ID: 424-87*D	Total length of sequence: 132806117 bp<br>Total number of sequences: 104494<br>Average contig length is: 1270 bp<br>Largest contig: 14862 bp<br>Shortest contig: 201 bp<br>N25 stats: 25% of total sequence length is contained in the 8367 sequences >= 2993 bp<br>N50 stats: 50% of total sequence length is contained in the 22020 sequences >= 2021 bp<br>N75 stats: 75% of total sequence length is contained in the 42561 sequences >= 1253 bp<br>Total GC count: 57846704 bp<br>GC %: 43.56 %
Saruma henryi	Living collections at The New York Botanical Garden. Rock Garden (Bronx, NY, USA).	Total length of sequence: 75298689 bp<br>Total number of sequences: 77163<br>Average contig length is: 975 bp<br>Largest contig: 14509 bp<br>Shortest contig: 201 bp<br>N25 stats: 25% of total sequence length is contained in the 5245 sequences >= 2620 bp<br>N50 stats: 50% of total sequence length is contained in the 14228 sequences >= 1711 bp<br>N75 stats: 75% of total sequence length is contained in the 28921 sequences >= 908 bp<br>Total GC count: 32959129 bp<br>GC %: 43.77 %

Table S2.

Accession numbers of *CRABS CLAW, SPATULA, NGATHA, and HECATE* homolog sequences used in this study.

CRABS CLAW sequences				
	**Outgroup**			
**Gene name**	**Species**	**Family**	**Accession number**	**Data Base**
AmtriCRC	*Amborella trichopoda*	Amborellaceae	evm_27.model.AmTr_v1.0 _scaffold00047.126	Phytozome
	**Basal Angiosperms**			
**Gene name**	**Species**	**Family**	**Accession number**	**Data Base**
AfimCRC	*Aristolochia fimbriata*	Aristolochiaceae	MN709133	NCBI
ArinCRC	*Aristolochia ringens*	Aristolochiaceae	MN709150	NCBI
AmanCRC	*Aristolochia manshuriensis*	Aristolochiaceae	MN709147	NCBI
AmacCRC	*Aristolochia macrophylla*	Aristolochiaceae	MN709143	NCBI
SaheCRC	*Saruma henryi*	Aristolochiaceae	MN709154	NCBI
AeurCRC	*Asarum europaeum*	Aristolochiaceae	MN709140	NCBI
	**Monocots**			
**Gene name**	**Species**	**Family**	**Accession number**	**Data Base**
MusaDL1	*Musa acuminata*	Musaceae	XM_009393202.2	NCBI
MusaDL2	*Musa acuminata*	Musaceae	XM_018826076.1	NCBI
MusaDL3	*Musa acuminata*	Musaceae	XM_009421461.2	NCBI
MusaDL4	*Musa acuminata*	Musaceae	XM_009407706.2	NCBI
OrzaDL	*Oryza sativa*	Poaceae	XM_015774589.2	NCBI
TritCRC	*Triticum aestivum*	Poaceae	AF545436.2	NCBI
SeitCRC	*Setaria italic*	Poaceae	XM_004985133.4	NCBI
SeviCRC	*Setaria viridis*	Poaceae	Sevir.9G494400.1	Phytozome
SbiCRC	*Sorghum bicolor*	Poaceae	XM_021451449.1	NCBI
ZemaDL	*Zea mays*	Poaceae	XM_008678090.3	NCBI
	**Basal Eudicots**			
**Gene name**	**Species**	**Family**	**Accession number**	**Data Base**
AquCRC	*Aquilegia coerulae*	Ranunculaceae	Aqcoe3G065300.1	Phytozome
EscaCRC	*Eschscholzia californica*	Papaveraceae	AM946412.1	NCBI
PsomCRC	*Papaver somniferum*	Papaveraceae	XM_026526584.1	NCBI
EpisaCRC	*Epimedium sagittatum*	Berberidaceae	JX560412.1	NCBI
ArmeCRC	*Argemone mexicana*	Papaveraceae		Phytometazyn
	**Core Eudicots**			
**Gene name**	**Species**	**Family**	**Accession number**	**Data Base**
CusaCRC	*Cucumis sativus*	Cucurbitaceae	XM_011657416.1	Phytozome
ViviCRC	*Vitis vinifera*	Vitaceae	GSVIVT01012246001	Phytozome
ThecaCRC	*Theobroma cacao*	Malvaceae	Thecc1EG011484t1	Phytozome
GoraCRC	*Gossypium raimondii*	Malvaceae	Gorai.005G146700.1	Phytozome
EugrCRC	*Eucalyptus grandis*	Myrtaceae	Eucgr.B03140.1	Phytozome
CicleCRC	*Citrus clementina*	Rutaceae	Ciclev10033770m	Phytozome
CapaCRC	*Carica papaya*	Caricaceae	evm.TU.contig_30608.1	Phytozome
FraveCRC	*Fragaria vesca*	Rosaceae	mrna04566.1-v1.0-hybrid	Phytozome
PrupeCRC	*Prunus persica*	Rosaceae	Prupe.1G290100.1	Phytozome
MadoCRC1	*Malus domestica*	Rosaceae	MDP0000291729	Phytozome

MadoCRC2	*Malus domestica*	Rosaceae	MDP0000171878	Phytozome
LiusCRC	*Linum usitatissimum*	Linaceae	Lus10000644	Phytozome
MetruCRC	*Medicago truncatula*	Fabaceae	Medtr5g046230.1	Phytozome
GlymaCRC1	*Glycine max*	Fabaceae	Glyma.02G121100.1	Phytozome
GlymaCRC2	*Glycine max*	Fabaceae	Glyma.01G063500.1	Phytozome
CleosCRC	*Cleome sparsifolia*	Cleomaceae	AY854803.1	NCBI
MiguCRC	*Mimulus guttatus*	Phrymaceae	Migut.E00040.1	Phytozome
BraraCRC	*Brassica rapa*	Brassicaceae	Brara.G02853.1	Phytozome
AthCRC	*Arabidopsis thaliana*	Brassicaceae	AT1G69180.1	TAIR
AlyrCRC	*Arabidopsis lyrata*	Brassicaceae	AL2G28270.t1	Phytozome
LeafCRC	*Lepidium africanum*	Brassicaceae	AY854802.1	NCBI
BoesCRC	*Boechera stricta*	Brassicaceae	Bostr.10273s0455.1	Phytozome
CaruCRC	*Capsella rubella*	Brassicaceae	Carubv10021641m	Phytozome
CagraCRC	*Capsella grandiflora*	Brassicaceae	Cagra.0537s0004.1	Phytozome
SolyCRC1	*Solanum lycopersicum*	Solanaceae	Solyc05g012050.2	Phytozome
SolyCRC2	*Solanum lycopersicum*	Solanaceae	Solyc01g010240.2.1	Phytozome
SotuCRC	*Solanum tuberosum*	Solanaceae	PGSC0003DMT400072893	Phytozome
NitaCRC	*Nicotiana tabacum*	Solanaceae	AY854800.1	NCBI
PetCRC	*Petunia x hybrida*	Solanaceae	AY854801.1	NCBI

SPATULA-ALCATRAZ sequences				
	**Outgroup**			
**Gene name**	**Species**	**Family**	**Accession number**	**Data Base**
CymicSPT	*Cycas micholitzii (Outgrup)*	Cycadaceae	XZUY-2049748	OneKP
	**Gymnosperms**			
**Gene name**	**Species**	**Family**	**Accession number**	**Data Base**
SunamSPT	*Sundacarpus amarus*	Podocarpaceae	KLGF-2092082	OneKP
TotaSPT	*Torreya taxifolia*	Taxaceae	EFMS-2015503	OneKP
	**Basal Angiosperms**			
**Gene name**	**Species**	**Family**	**Accession number**	**Data Base**
AmtrSPT	*Amborella trichopoda*	Amborellaceae	URDJ-2004099	OneKP
AuscSPT	*Austrobaileya scandens*	Austrobaileyaceae	FZJL-2164770	OneKP
SarglSPT	*Sarcandra glabra*	Chloranthaceae	QSHQ-2009866	OneKP
AscruSPT	*Ascarina rubricaulis*	Chloranthaceae	WZFE-2194308	OneKP
AfimSPT	*Aristolochia fimbriata*	Aristolochiaceae	MN709134	NCBI
AeleSPT	*Aristolochia elegans*	Aristolochiaceae	PAWA-2009790	OneKP
ArinSPT	*Aristolochia ringens*	Aristolochiaceae	MN709151	NCBI
AarSPT	*Aristolochia arborea*	Aristolochiaceae	MN709136	NCBI
AmanSPT	*Aristolochia manshuriensis*	Aristolochiaceae	MN709148	NCBI
AmacSPT	*Aristolochia macrophylla*	Aristolochiaceae	MN709144	NCBI
	**Monocots**			
**Gene name**	**Species**	**Family**	**Accession number**	**Data Base**
HydeSPT	*Hypoxis decumbens*	Hypoxidaceae	KY421366	NCBI
LepgiSPT	*Lepidosperma gibsonii*	Cyperaceae	WBIB-2064717	OneKP
ZemaSPT1	*Zea mays*	Poaceae	GRMZM2G017349	Phytozome
ZemaSPT2	*Zea mays*	Poaceae	GRMZM2G030744	Phytozome

SobiSPT1	*Sorghum bicolor*	Poaceae	Sb10g004500	Phytozome
SobiSPT2	*Sorghum bicolor*	Poaceae	Sb04g036450	Phytozome
OrysaSPT1	*Oryza sativa*	Poaceae	LOC-Os06g06900	Phytozome
OrysaSPT2	*Oryza sativa*	Poaceae	LOC-Os02g56140	Phytozome
	**Basal Eudicots**			
**Gene name**	**Species**	**Family**	**Accession number**	**Data Base**
AquSPT	*Aquilegia coerulea*	Ranunculaceae	Aquca-007-007-02	Phytozome
BofrSPT1	*Bocconia frutescens*	Papaveraceae	KY421363	NCBI
BofrSPT2	*Bocconia frutescens*	Papaveraceae	KY421364	NCBI
BofrSPT3	*Bocconia frutescens*	Papaveraceae	KY421365	NCBI
ArgmeSPT	*Argemone mexicana*	Papaveraceae	IRAF-2111869	OneKP
ParhoSPT	*Papaver rhoeas*	Papaveraceae	MVTX-2101636	OneKP
PasetSPT	*Papaver setigerum*	Papaveraceae	MLPX-2019231	OneKP
PsomSPT	*Papaver somniferum*	Papaveraceae	RQNK-2019222	OneKP
EscaSPT	*Eschscholzia californica*	Papaveraceae	EVOD-2110824	OneKP
	**Core Eudicots**			
**Gene name**	**Species**	**Family**	**Accession number**	**Data Base**
FraveSPT	*Fragaria vesca*	Rosaceae		Phytozome
PpSPT	*Prunus persica*	Rosaceae	Prupe.7G131400.1	Phytozome
AthaSPT	*Arabidopsis thaliana*	Brassicaceae	AT4G36930	Phytozome
AlyrSPT	*Arabidopsis lyrata*	Brassicaceae	353119	Phytozome
BraraSPT1	*Brassica rapa*	Brassicaceae	Bra011740	Phytozome
BraraSPT2	*Brassica rapa*	Brassicaceae	Bra010591	Phytozome
SotuSPT	*Solanum tuberosum*	Solanaceae	PGSC0003DMT400010253	Phytozome
SolySPT	*Solanum lycopersicum*	Solanaceae	Solyc02g093280.2	Phytozome
ViviALC	*Vitis vinifera*	Vitaceae	GSVIVT01022111001	Phytozome
BraraALC	*Brassica rapa*	Brassicaceae	Brara.G01197.1	Phytozome
BraolALC	*Brassica oleraceae*	Brassicaceae	Bol027228	Phytozome
AthaSPT	*Arabidopsis thaliana*	Brassicaceae	AT5G67110	TAIR
AlyrSPT	*Arabidopsis lyrata*	Brassicaceae	AL8G44250.t1	Phytozome
CapruALC	*Capsella rubella*	Brassicaceae	Carubv10028570m	Phytozome
CagraALC	*Capsella grandiflora*	Brassicaceae	Cagra.2428s0008.1	Phytozome

NGATHA sequences				
	**Outgroup**			
**Gene name**	**Species**	**Family**	**Accession number**	**Data Base**
AtRAV1	*Arabidopsis thaliana*	Brassicaceae	AT1G13260.1	TAIR
	**Basal Angiosperms**			
**Gene name**	**Species**	**Family**	**Accession number**	**Data Base**
AfimNGA	*Aristolochia fimbriata*	Aristolochiaceae	MN709130	NCBI
ArelNGA	*Aristolochia elegans*	Aristolochiaceae	PAWA-2008421	OneKP
SaheNGA	*Saruma henryi*	Aristolochiaceae	QDVW-2013083	OneKP
ArinNGA	*Aristolochia ringens*	Aristolochiaceae	MN709149	NCBI
AarNGA	*Aristolochia arborea*	Aristolochiaceae	MN709135	NCBI
AmacNGA	*Aristolochia macrophylla*	Aristolochiaceae	MN709141	NCBI
AeurNGA	*Asarum europaeum*	Aristolochiaceae	MN709137	NCBI
	**Monocots**			
**Gene name**	**Species**	**Family**	**Accession number**	**Data Base**

OryNGA1	*Oryza sativa*	Poaceae	LOC_Os03g02900.1	Phytozome
OryNGA2	*Oryza sativa*	Poaceae	LOC_Os02g45850.1	Phytozome
OryNGA3	*Oryza sativa*	Poaceae	LOC_Os12g06080.1	Phytozome
OryNGA4	*Oryza sativa*	Poaceae	LOC_Os11g05740.1	Phytozome
BradNGA1	*Brachypodium distachyon*	Poaceae	Bradi5g19260.2	Phytozome
BradNGA2	*Brachypodium distachyon*	Poaceae	Bradi3g51840.3	Phytozome
BradNGA3	*Brachypodium distachyon*	Poaceae	Bradi2g47220.1	Phytozome
BradNGA4	*Brachypodium distachyon*	Poaceae	Bradi1g77150.1	Phytozome
BradNGA5	*Brachypodium distachyon*	Poaceae	Bradi4g25170.1	Phytozome
BradNGA6	*Brachypodium distachyon*	Poaceae	Bradi3g32140.1	Phytozome
BrasNGA1	*Brachypodium stacei*	Poaceae	Brast09G182000.1	Phytozome
BrasNGA2	*Brachypodium stacei*	Poaceae	Brast04G103000.1	Phytozome
BrasNGA3	*Brachypodium stacei*	Poaceae	Brast02G019600.1	Phytozome
BrasNGA4	*Brachypodium stacei*	Poaceae	Brast03G184100.1	Phytozome
BrasNGA5	*Brachypodium stacei*	Poaceae	Brast01G176300.1	Phytozome
BrasNGA6	*Brachypodium stacei*	Poaceae	Brast05G016300.1	Phytozome
BrasNGA7	*Brachypodium stacei*	Poaceae	Brast07G005200.1	Phytozome
	**Basal Eudicots**			
**Gene name**	**Species**	**Family**	**Accession number**	**Data Base**
AqcoeNGA1	*Aquilegia coerulea*	Ranunculaceae	Aqcoe5G472800.1	Phytozome
EschcaNGA1	*Eschscholzia californica*	Papaveraceae	EVOD-2110824	OneKP
NenuNGA1	*Nelumbo nucifera*	Nelumbonaceae	XM_010252998.2	NCBI
NenuNGA2	*Nelumbo nucifera*	Nelumbonaceae	XM_010242757.2	NCBI
	**Core Eudicots**			
**Gene name**	**Species**	**Family**	**Accession number**	**Data Base**
NitabNGA1	*Nicotiana tabacum*	Solanaceae	XM_016610765.1	NCBI
NitabNGA2	*Nicotiana tabacum*	Solanaceae	XM_016641154.1	NCBI
NitabNGA3	*Nicotiana tabacum*	Solanaceae	XM_016655383.1	NCBI
NitabNGA4	*Nicotiana tabacum*	Solanaceae	XM_016656424.1	NCBI
SoltuNGA1	*Solanum tuberosum*	Solanaceae	XM_006343694.2	NCBI
SoltuNGA2	*Solanum tuberosum*	Solanaceae	XM_006352159.2	NCBI
SoltuNGA3	*Solanum tuberosum*	Solanaceae	XM_006352237.2	NCBI
SolyNGA1	*Solanum lycopersicum*	Solanaceae	Solyc08g083400.2.1	Phytozome
SolyNGA3	*Solanum lycopersicum*	Solanaceae	Solyc08g013700.1.1	Phytozome
AtNGA1	*Arabidopsis thaliana*	Brassicaceae	AT2G46870	TAIR
AtNGA2	*Arabidopsis thaliana*	Brassicaceae	AT3G61970	TAIR
AtNGA3	*Arabidopsis thaliana*	Brassicaceae	AT1G01030	TAIR
AtNGA4	*Arabidopsis thaliana*	Brassicaceae	AT4G01500	TAIR
AlyrNGA1	*Arabidopsis lyrata*	Brassicaceae	AL181U10040.t1	Phytozome
AlyrNGA2	*Arabidopsis lyrata*	Brassicaceae	AL4G46330.t1	Phytozome
AlyrNGA3	*Arabidopsis lyrata*	Brassicaceae	AL5G44580.t1	Phytozome
AlyrNGA4	*Arabidopsis lyrata*	Brassicaceae	AL1G11500.t1	Phytozome
AlyrNGA5	*Arabidopsis lyrata*	Brassicaceae	AL6G51880.t1	Phytozome
CapruNGA1	*Capsella rubella*	Brassicaceae	Carubv10023656m	Phytozome
CapruNGA2	*Capsella rubella*	Brassicaceae	Carubv10017696m	Phytozome
CapruNGA3	*Capsella rubella*	Brassicaceae	Carubv10009540m	Phytozome
CapruNGA4	*Capsella rubella*	Brassicaceae	Carubv10001406m	Phytozome

HECATE sequences				
	**Outgroup**			
**Gene name**	**Species**	**Family**	**Accession number**	**Data Base**
AtbHLH87	Arabidopsis thaliana	Brassicaceae	AF488617.1	NCBI
	**Basal Angiosperms**			
**Gene name**	**Species**	**Family**	**Accession number**	**Data Base**
AmtrHEC1	*Amborella trichopoda*	Amborellaceae	XM_006827995.3	NCBI
AmtrHEC2	*Amborella trichopoda*	Amborellaceae	XM_006852430.2	NCBI
AmtrHEC3	*Amborella trichopoda*	Amborellaceae	XM_006858410.3	NCBI
AfimHEC1	*Aristolochia fimbriata*	Aristolochiaceae	MN709131	NCBI
AfimHEC3	*Aristolochia fimbriata*	Aristolochiaceae	MN709132	NCBI
AmacHEC1	*Aristolochia macrophylla*	Aristolochiaceae	MN709142	NCBI
AmanHEC1	*Aristolochia manshuriensis*	Aristolochiaceae	MN709146	NCBI
AeurHEC1	*Asarum europaeum*	Aristolochiaceae	MN709138	NCBI
AeurHEC3	*Asarum europaeum*	Aristolochiaceae	MN709139	NCBI
SaheHEC1	*Saruma henryi*	Aristolochiaceae	MN709153	NCBI
	**Monocots**			
**Gene name**	**Species**	**Family**	**Accession number**	**Data Base**
OrothHEC1	*Oropetium thomaeum*	Poaceae	Oropetium_20150105_05690A	Phytozome
OrothHEC2	*Oropetium thomaeum*	Poaceae	Oropetium_20150105_20418A	Phytozome
OrothHEC3	*Oropetium thomaeum*	Poaceae	Oropetium_20150105_13681A	Phytozome
OrysaHEC1	*Oryza sativa*	Poaceae	LOC_Os08g36740.1	Phytozome
OrysaHEC1-2	*Oryza sativa*	Poaceae	LOC_Os08g36740.1	Phytozome
OrysaHEC2	*Oryza sativa*	Poaceae	LOC_Os08g01700.1	Phytozome
OrysaHEC3	*Oryza sativa*	Poaceae	LOC_Os09g28210.1	Phytozome
	**Basal Eudicots**			
**Gene name**	**Species**	**Family**	**Accession number**	**Data Base**
AqcoeHEC1	*Aquilegia coerulea*	Ranunculaceae	Aqcoe6G096700.1	Phytozome
AqcoeHEC2	*Aquilegia coerulea*	Ranunculaceae	Aqcoe6G096700.2	Phytozome
AqcoeHEC3	*Aquilegia coerulea*	Ranunculaceae	Aqcoe5G239900.1	Phytozome
NenuHEC1	*Nelumbo nucifera*	Nelumbonaceae	LOC104601763	Phytozome
NenuHEC2	*Nelumbo nucifera*	Nelumbonaceae	LOC104606174	Phytozome
NenuHEC3	*Nelumbo nucifera*	Nelumbonaceae	LOC104589283	Phytozome
	**Core Eudicots**			
**Gene name**	**Species**	**Family**	**Accession number**	**Data Base**
AthaHEC1	*Arabidopsis thaliana*	Brassicaceae	AT5G67060	TAIR
AthaHEC2	*Arabidopsis thaliana*	Brassicaceae	AT3G50330	TAIR
AthaHEC3	*Arabidopsis thaliana*	Brassicaceae	AT5G09750	TAIR
AlyrHEC1	*Arabidopsis lyrata*	Brassicaceae	XM_021012347.1	Phytozome
AlyrHEC2	*Arabidopsis lyrata*	Brassicaceae	XM_021012353.1	Phytozome
AlyrHEC3	*Arabidopsis lyrata*	Brassicaceae	XM_021023153.1	Phytozome
CapruHEC1	*Capsella rubella*	Brassicaceae	XM_006281680.2	Phytozome
CapruHEC2	*Capsella rubella*	Brassicaceae	XM_006292743.1	Phytozome
CapruHEC3	*Capsella rubella*	Brassicaceae	XM_006290147.2	Phytozome

EucgHEC1	*Eucalyptus grandis*	Myrtaceae	Eucgr.H01682.1	Phytozome
EucgHEC2	*Eucalyptus grandis*	Myrtaceae	XM_010028135.1	NCBI
EucgHEC3	*Eucalyptus grandis*	Myrtaceae	XM_010029062.1	NCBI
VitvHEC1	*Vitis vinifera*	Vitaceae	XM_002277814.3	NCBI
VitvHEC3	*Vitis vinifera*	Vitaceae	XM_002275105.3	NCBI

Table S3. Primers used in this study. Gene specific primers used in this study to amplify *AfimCRC, AfimSPT, AfimNGA*, and *AfimHEC1,* and *AfimHEC3* orthologs in *Aristolochia fimbriata*, using reverse transcription polymerase chain reaction (RT-PCR), quantitative reverse transcription (qRT-PCR), and *in situ* hybridization (ISH) experiments.

**Primer name (RT-PCR)**	**Primer sequence (5´ - 3´)**
AfimCRC Fwd	ATGAAAATTGGAGGCTTTTCC
AfimCRC Rev	GGATCCATCAGTACCCATGTGATC
AfimSPT Fwd	GAATTCATGTACGGGAAGATATGCTCG
AfimSPT Rev	GGATCCTCAAAATTTTAATGCTTC
AfimNGA Fwd	CCATGGACAGTAGTTGTATCGAGG
AfimNGA Rev	GGATCCTTACAAGGCTCCGACACA
AfimHEC1 Fwd	CATATGGAAGGTCACGAGCTGGTA
AfimHEC1 Rev	GAATTCTCAGCTGAGCAAGTGGGT
AfimHEC3 Fwd	CATATGAAGGAAATGATGTACAAA
AfimHEC3 Rev	GAATTCTCACTGATGTTTCATTAA
AfimACTINA Fwd	GGTTGGGATGGGTCAGAAGG
AfimACTINA Rev	ACTTGCCCATCAGGAAGCTC
**Primer name (qRT-PCR)**	
AfimCRC Fwd	GAAATGTGGCCACTGTGGTA
AfimCRC Rev	GCTGGGTAGTCGTTGGAGAA
AfimSPT Fwd	AGTGTCCACTTCTGATAGGCA
AfimSPT Rev	CTCTCCCACTTCGCTCTCAT
AfimNGA Fwd	AGCAGCTCTACATCGACTGG
AfimNGA Rev	GCTCCGACACATTGCTTCTT
AfimUBC Fwd	TGGGTCCTCCTGATAGTCCA
AfimUBC Rev	AGAGATGGTGAGTGCTGGAC
**Primername (ISH)**	
AfimCRC Fwd	TGTCTGGCCATGGAGTTC
AfimCRC Rev	CTTAATACGACTCACTATAGGGCTGGAGGCTTCTCCTCAT
AfimSPT Fwd	CAAATGTCCCAGATGCGCATA
AfimSPT Rev	CTTAATACGACTCACTATAGGGGTGTCCCTTCCCAGAGTG
AfimNGA Fwd	GTATCGCCGGCGTTGAGT
AfimNGA Rev	CTTAATACGACTCACTATAGGGCTCGTCTTCCGTCTCCGA
AfimHEC1 Fwd	TTGTAGTGTCTACGACTCCATCC
AfimHEC1 Rev	CTTAATACGACTCACTATAGGGCGCCCAATTCCGTTACACTA
AfimHEC3 Fwd	GCCACCATGCTTGACGAA
AfimHEC3 Rev	CTTAATACGACTCACTATAGGGTAATTCGTAGTCTTGGTGGTG

Figure S1. Maximum likelihood (ML) phylogenetic analysis of the *CRC* gene lineage in flowering plants. The outgroup used was the *Amborella trichopoda AmtrCRC*. Bootstrap (BS) supports shown above the nodes. Colors follow conventions on the figure.

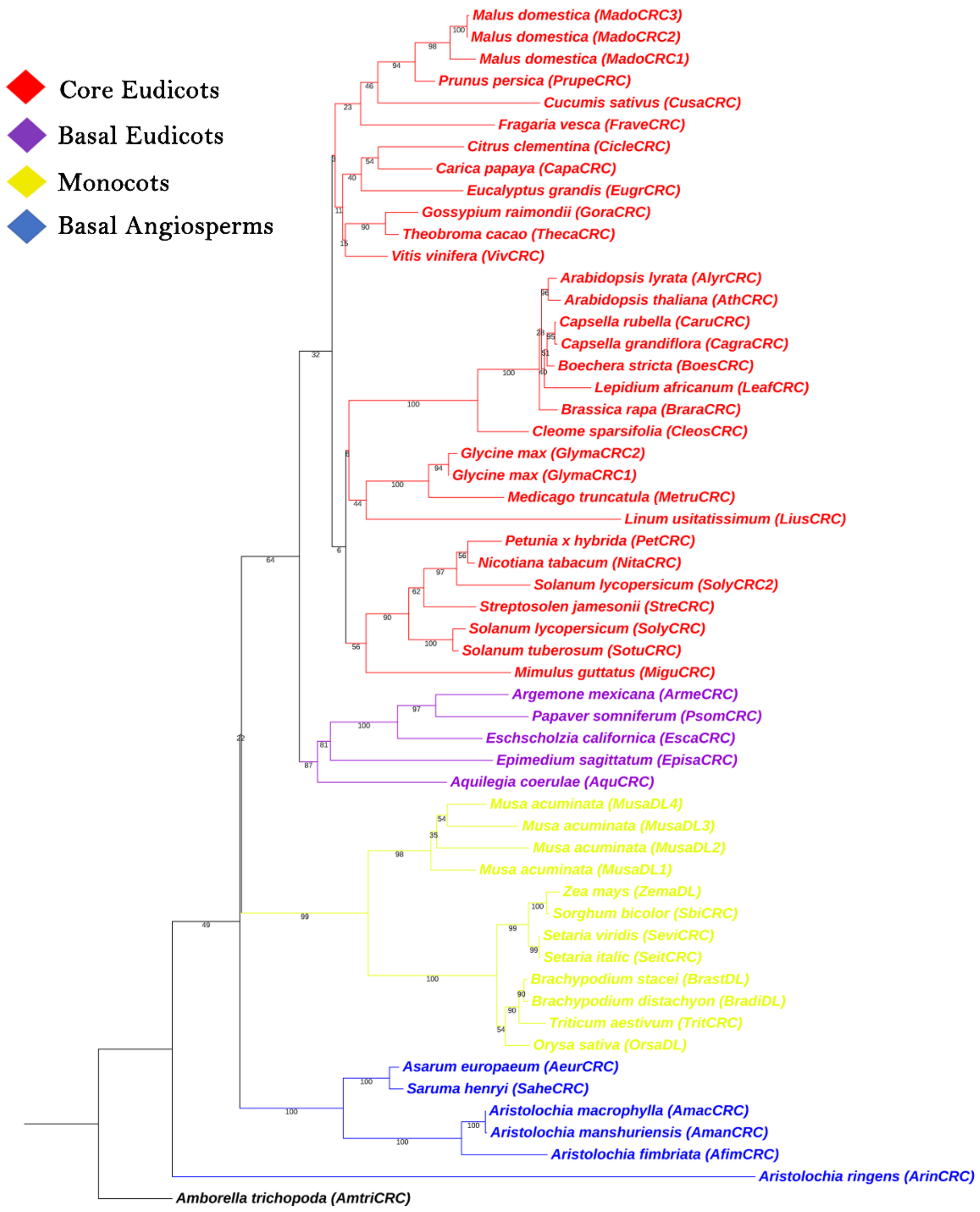

Figure S2. Maximum likelihood (ML) phylogenetic analysis of the *SPT* gene lineage in flowering plants. Outgroup used was *Cycas micholitzii SPT*. Star points to a duplication event. BS supports shown in the nodes. Colors follow conventions on the figure.

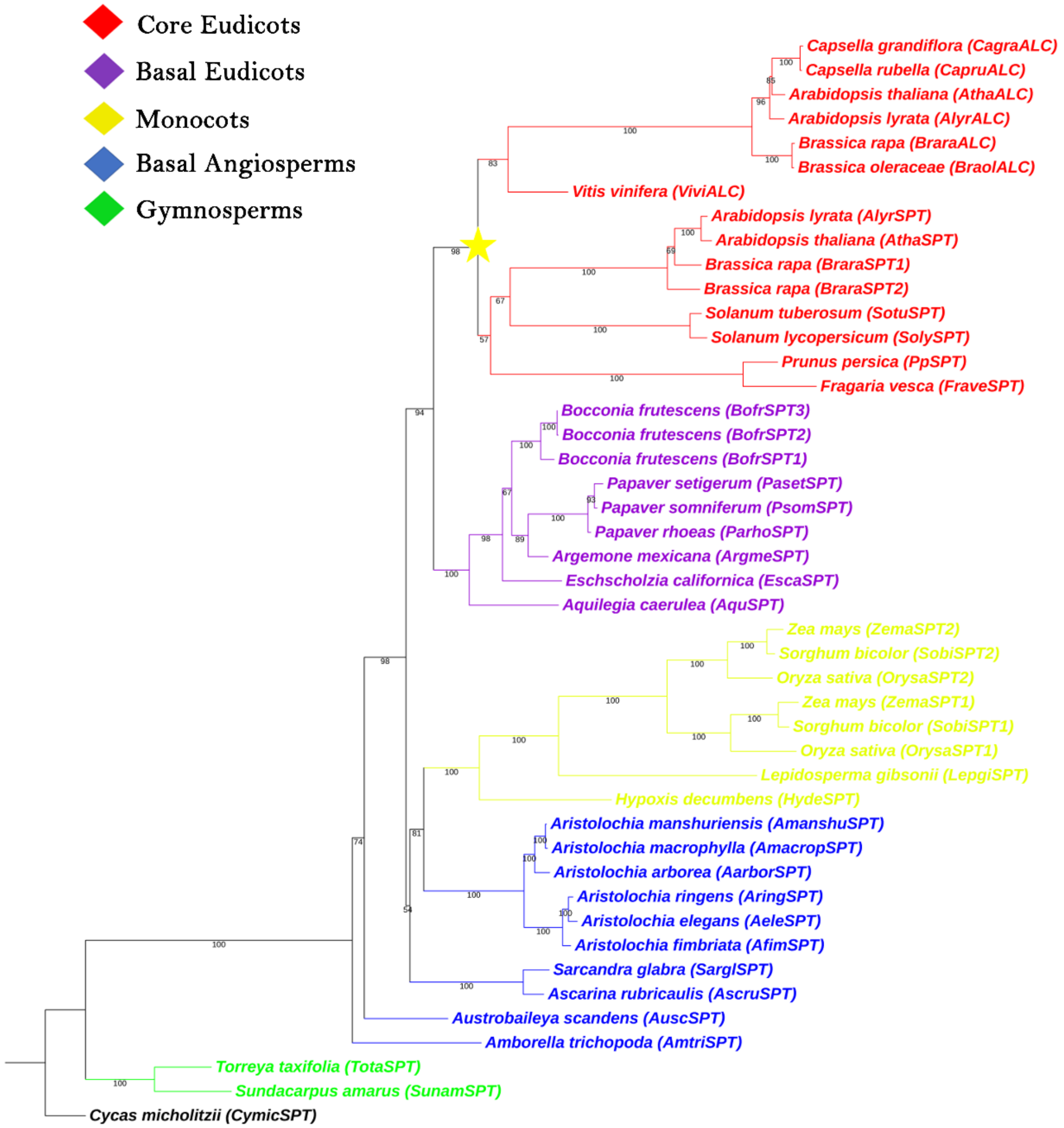

Figure S3. Maximum likelihood (ML) phylogenetic analysis of the *NGA* gene lineage in flowering plants. The outgroup used was *Arabidopsis thaliana RAV*. Star points to a duplication event. BS supports shown in the nodes. Colors follow conventions on the figure.

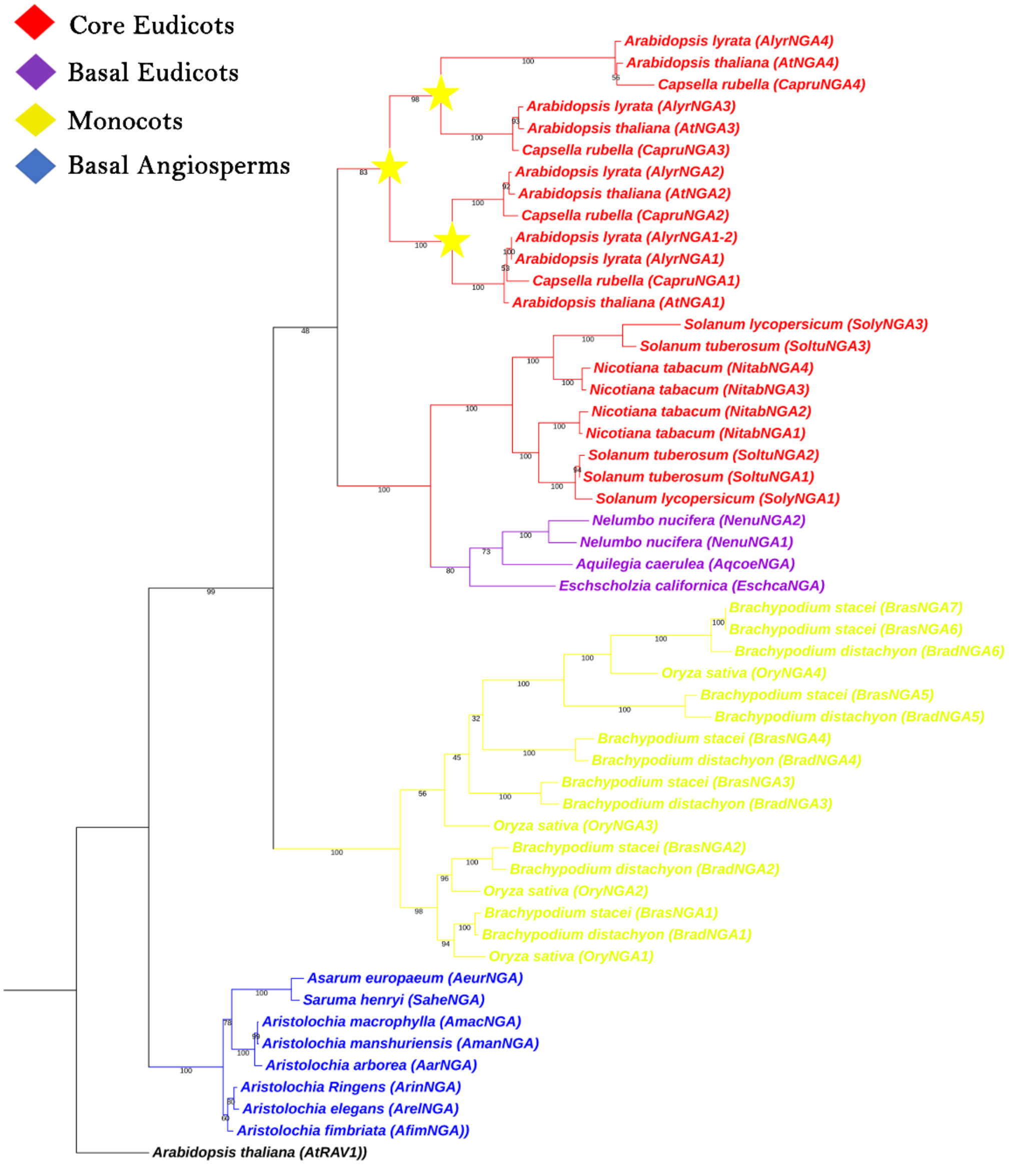

Figure S4. Maximum likelihood (ML) phylogenetic analysis of the *HEC* gene lineage in flowering plants. Outgroup used was *Arabidopsis thaliana bHLH87.* Star points to a duplication event. BS supports shown in the nodes. Colors follow conventions on the figure.

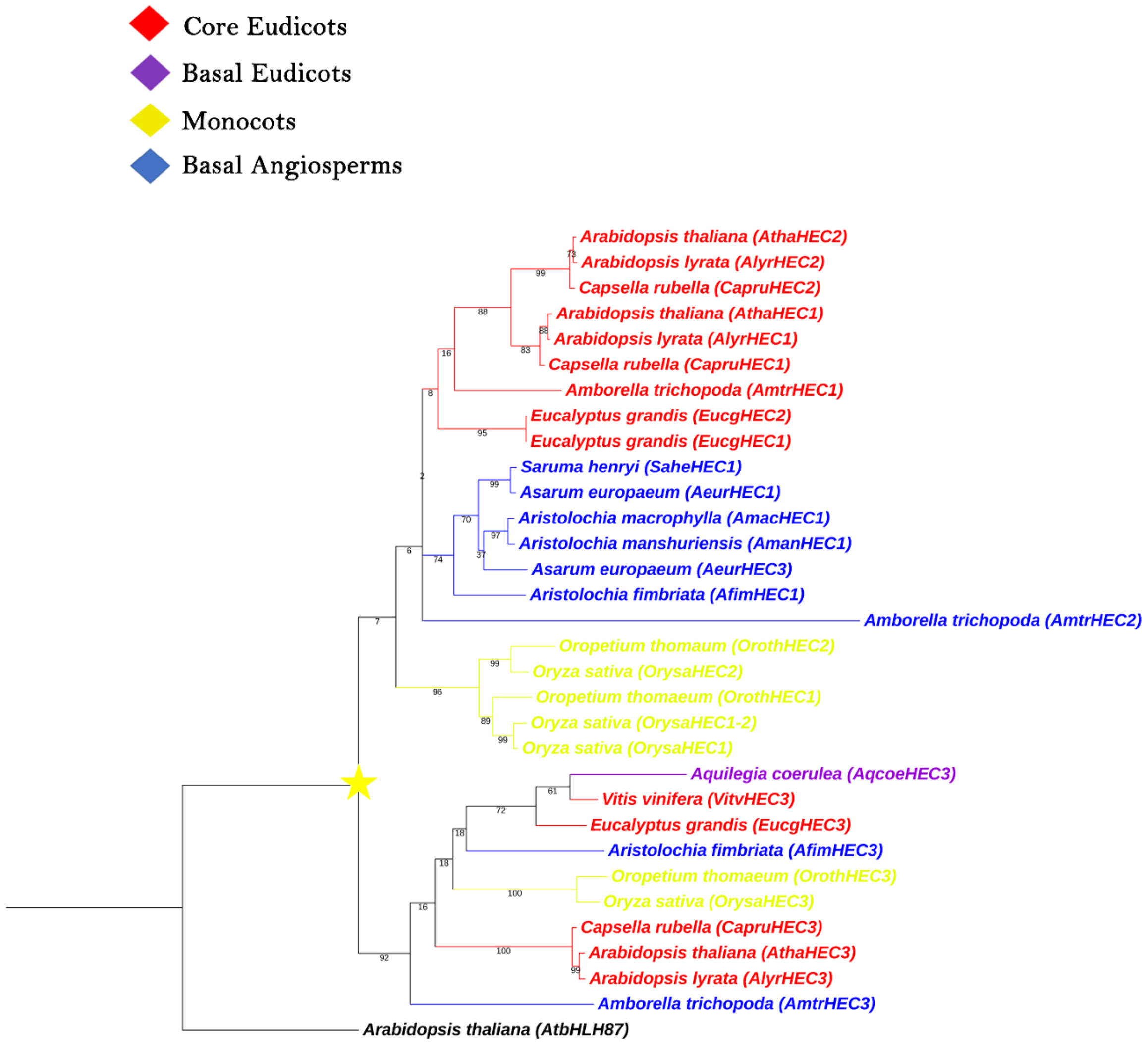

Figure S5. Relative expression profiles of *AfimCRC*, *AfimSPT* and *AfimNGA* at different floral developmental stages, and leaves using standard qRT-PCR. Ubiquitin (*AfimUBC*) was used as a positive control. Abbreviations: **S5**, stage 5 of the floral bud, when stigmatic lobes are fully developed. **S7**, stage 7 of the floral bud, when the stigmatic lobes grow above the anthers and the ovules initiate to develop. **S9**, stage 9 of the floral bud, when ovules develop the two integuments. **L**, leaf; **A**, Shoot apex with early floral buds (developmental stages S1- S4); **Ov**, Ovary; **Gy,** gynostemium**; -C** indicates the amplification reaction loaded without cDNA.

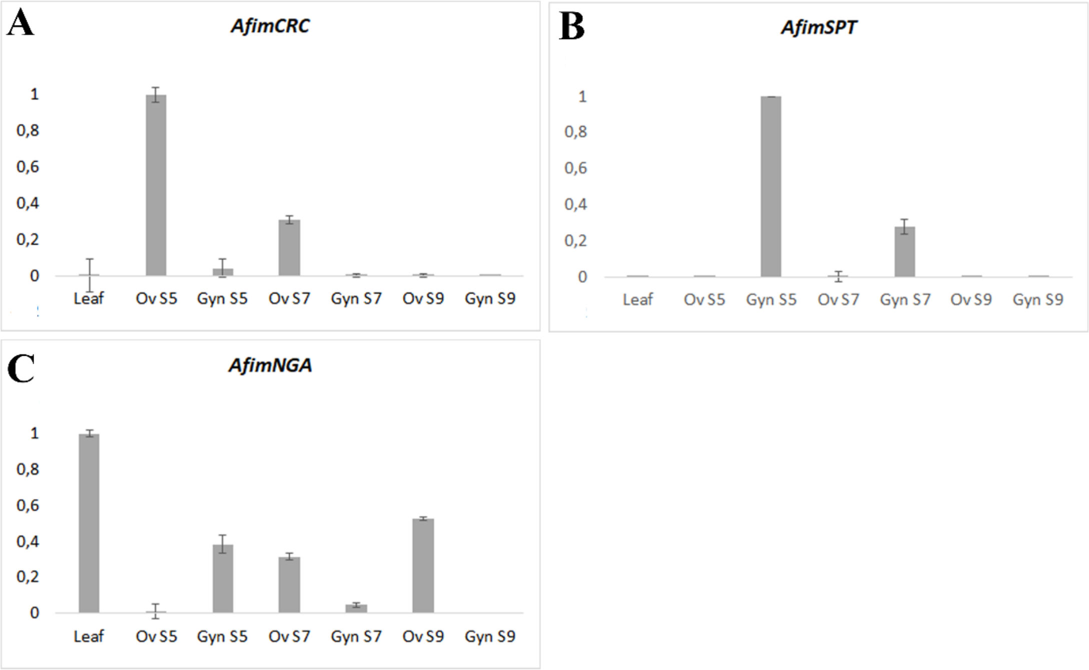

Figure S6. *In situ* hybridization with sense probes for all tested genes in *Aristolochia fimbriata* resulted in no signal. **A** *AfimCRC* in floral bud at S5 **B** *AfimNGA* in transverse section of a gynostemium at S9 **C** *AfimSPT* in a floral bud at S4 **D** *AfimHEC1* in flowering shoot apex in longitudinal section **E** *AfimHEC3* in flowering shoot apex in longitudinal section. Abbreviations: **ab**, accessory bud; **l**, leaf; **ov,** ovary; **p,** perianth. Black arrows (**D**, **E**) points to the shoot apical meristem; black arrowheads (**A-C**) point to stamens or stamen primordia; asterisks (*) (**A**, **C**) point to stigmatic lobes. White arrows (**A**) indicate the putative style. Scale bars: 40 µm in **A, B**; 100 µm in **C, E**; **200** µm in **D**.

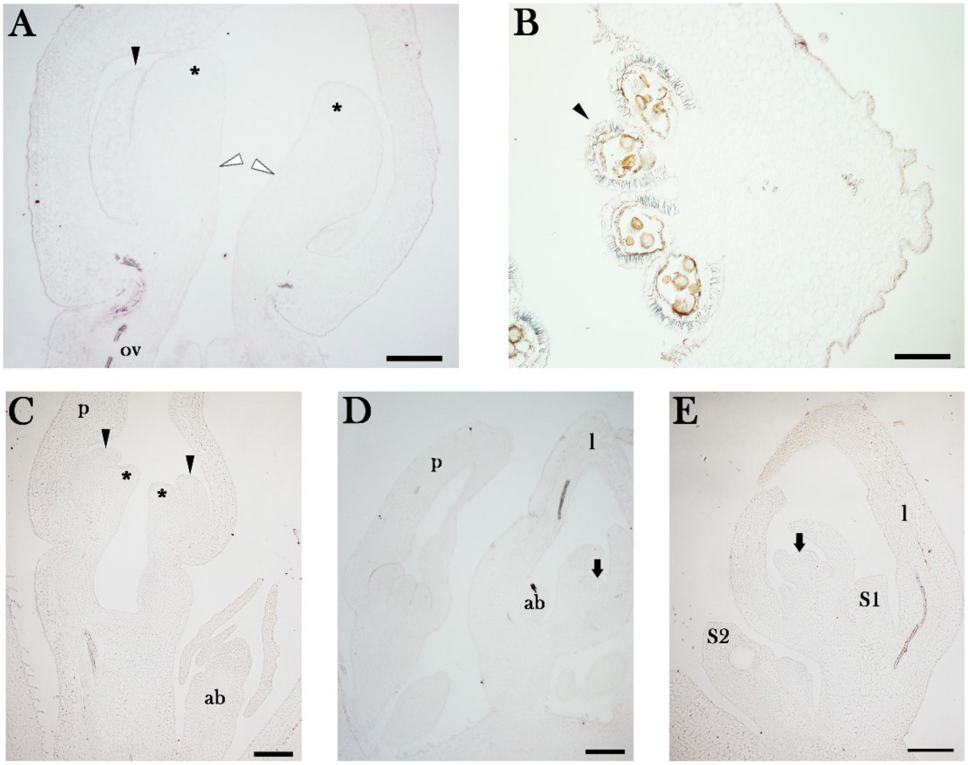

Supplement: Supplementary file 1 — Additional file 1: Table 1. Plant collections, locations, and statistics from the TRINITY assembly of mixed transcriptomes obtained for each of the members of the perianth-bearing Piperales. Table S2. Accession numbers of CRABS CLAW, SPATULA, NGATHA, and HECATE homolog sequences used in this study. Table S3. Primers used in this study. Gene specific primers used in this study to amplify AfimCRC, AfimSPT, AfimNGA, and AfimHEC1, and AfimHEC3 orthologs in Aristolochia fimbriata, using reverse transcription polymerase chain reaction (RT-PCR), quantitative reverse transcription (qRT-PCR), and in situ hybridization (ISH) experiments. Figure S1. Maximum likelihood (ML) phylogenetic analysis of the CRC gene lineage in flowering plants. The outgroup used was the Amborella trichopoda AmtrCRC. Bootstrap (BS) supports shown above the nodes. Colors follow conventions on the figure. Figure S2. Maximum likelihood (ML) phylogenetic analysis of the SPT gene lineage in flowering plants. Outgroup used was Cycas micholitzii SPT. Star points to a duplication event. BS supports shown in the nodes. Colors follow conventions on the figure. Figure S3. Maximum likelihood (ML) phylogenetic analysis of the NGA gene lineage in flowering plants. The outgroup used was Arabidopsis thaliana RAV. Star points to a duplication event. BS supports shown in the nodes. Colors follow conventions on the figure. Figure S4. Maximum likelihood (ML) phylogenetic analysis of the HEC gene lineage in flowering plants. Outgroup used was Arabidopsis thaliana bHLH87. Star points to a duplication event. BS supports shown in the nodes. Colors follow conventions on the figure. Figure S5. Relative expression profiles of AfimCRC, AfimSPT and AfimNGA at different floral developmental stages, and leaves using standard qRT-PCR. Ubiquitin (AfimUBC) was used as a positive control. Figure S6. In situ hybridization with sense probes for all tested genes in Aristolochia fimbriata. [file 13227_2020_149_MOESM1_ESM.pdf]
